# Factors and Psychosocial and Physical Health Outcomes Associated with Cyberbullying Victimization in Adolescents: A Systematic Scoping Review

**DOI:** 10.3390/bs16101733

**Published:** 2026-09-23

**Authors:** Adrián Navarro-Vitoria, Rosa M. Patró-Hernández, Carmen M. Galvez-Sánchez, Rosa M. Limiñana-Gras

**Affiliations:** 1 Department of Personality, Evaluation and Psychological Treatment, Faculty of Psychology and Speech Therapy, University of Murcia, Building 31, 30100 Murcia, Spain; adrian.navarrov@um.es (A.N.-V.); rosapatro@um.es (R.M.P.-H.); liminana@um.es (R.M.L.-G.); 2 Regional International Campus of Excellence (CEIR), Mare Nostrum Campus (CMN), 30100 Murcia, Spain; 3 Assisted Reproduction Unit, QuironSalud Murcia Medical Center, 30008 Murcia, Spain

**Keywords:** cyberbullying, cybervictims, associated factors, health outcomes, adolescents, victims

## Abstract

**Background/Objectives**: Cyberbullying victimization is a form of online harassment that can adversely affect adolescents’ psychological, social, and physical well-being. Although research on cyberbullying has increased substantially in recent years, the factors associated with cyberbullying victimization and its medium- and long-term psychosocial consequences remain insufficiently integrated within the literature. This systematic scoping review aimed to (1) identify factors associated with adolescent cyberbullying victimization across individual, family, school/peer, and online-context levels and (2) synthesize the associated psychosocial and physical health outcomes. **Methods**: The review focused on secondary-school adolescents, with 12–17 years as the target age range. Studies identifying participants as secondary-school students without reporting exact age ranges were also eligible. This systematic scoping review was conducted in accordance with the Joanna Briggs Institute (JBI) methodology for scoping reviews and reported following the Preferred Reporting Items for Systematic Reviews and Meta-Analyses Extension for Scoping Reviews (PRISMA-ScR). The review protocol was prospectively registered in PROSPERO and the Open Science Framework. A literature search was conducted through three platforms—EBSCOhost, Web of Science, and ProQuest—using the databases and indexes available within each platform. The methodological quality of the included studies was also appraised. **Results**: Of the 697 records identified, 24 studies met the eligibility criteria and were included in the review. Methodological quality assessment classified four studies as high quality, thirteen as moderate quality, and seven as low quality. Factors associated with cyberbullying victimization were organized across individual (psychological characteristics and substance use), family (family circumstances and parental phubbing), school/peer (offline peer victimization), and online-context (internet and social media use and personal information sharing) levels. Associated outcomes encompassed psychological and emotional difficulties, behavioral problems, social and academic difficulties, and poorer physical health. Parental support and parent–child bonding were examined as modifiers of associations with individual outcomes. **Conclusions**: Cyberbullying victimization among adolescents is associated with multiple individual and contextual factors and with a broad range of adverse psychosocial and physical health outcomes. The findings highlight the need for multilevel prevention strategies that address personal, family, peer, school, and digital-context factors, alongside accessible support systems for affected adolescents. Further longitudinal research is needed to clarify temporal relationships between cyberbullying victimization, associated individual and contextual factors, and psychosocial and physical health outcomes.

## 1. Introduction

The expansion of information and communication technologies (ICT) has simplified daily life and transformed both lifestyles and patterns of communication (Marinoni et al., 2026). This shift has had a particularly marked impact on adolescents, who increasingly rely on social media as a primary means of staying connected with their peers. However, these technologies have also become tools that facilitate access to minors and enable peer bullying, thereby increasing the risk that adolescents become victims of online aggression (Bazaga, 2022).

Cyberbullying, a form of bullying that occurs through the internet or social media, has extended beyond the socio-educational sphere and now permeates a wide range of online platforms. Victims frequently face ongoing humiliation and harassment and often lack the social skills and personal resources needed to respond assertively (Garaigordobil, 2018). Cyberbullying can be defined as an intentional and repeated form of aggressive behavior perpetrated by an individual or group through digital technologies, with the purpose of inflicting harm on a victim who is unable to defend themselves easily. It constitutes a misuse of electronic media to exert online social aggression and is typically characterized by anonymity, repetition, and a clear power imbalance between aggressors and victims (Smith et al., 2008; Vismara et al., 2022). A global prevalence review by C. Zhu et al. (2021) reports cyberbullying victimization rates ranging from 14.6% to 52.2%, and perpetration rates from 6.3% to 32% across several countries.

In this review, cyberbullying victimization refers to experiencing cyberbullying as its target, whereas perpetration refers to engaging in cyberbullying against others. “Cyberbullying involvement” is a broader term that may encompass victimization, perpetration, or both, depending on the primary study. Bully–victim status refers to participants classified as both experiencing and perpetrating cyberbullying within the period assessed. Such overlap between roles does not, by itself, establish a temporal transition or a reciprocal relationship.

The accessibility and permanence of humiliating content, together with the continuous nature of online harassment, are distinctive characteristics of cyberbullying. Cyberbullying can occur at any time of day and persist over extended periods (Vismara et al., 2022; C. Zhu et al., 2021). Cyberbullying victimization has been associated with internalizing difficulties, including anxiety, depression, and low self-esteem (Garaigordobil & Navarro, 2022; Guo, 2016; Lei et al., 2020; Marciano et al., 2020; Molero et al., 2022; Tran et al., 2023; J. Zhu et al., 2021). Limited social support and peer rejection have also been examined in relation to cybervictimization (Guo, 2016; Holfeld & Leadbeater, 2017; Worsley et al., 2019), alongside feelings of defenselessness, negative self-concept, and low self-esteem (Guo, 2016; Lei et al., 2020; Luengo Latorre, 2019; Navarro et al., 2018; Ortega-Barón & Carrascosa, 2018). Adolescence, particularly in the middle and late stages (approximately 12–17 years), is a developmental period characterized by increasing autonomy in digital media use, expanding social networks, and intense peer relationships. The increased time spent online during secondary education on social media and other communication platforms amplifies both opportunities for connection and exposure to peer-related risks such as cyberbullying victimization. Focusing specifically on adolescents in secondary education is therefore crucial, as cyberbullying experiences during this stage coincide with critical transitions in identity formation, emotional regulation, and academic adjustment, and may have lasting implications for psychosocial health and well-being. For this reason, the present review deliberately restricts its scope to adolescents, rather than combining children, younger students, or university populations.

Several reviews have already examined cyberbullying among young people, addressing issues such as prevalence rates, correlates of involvement, and specific psychological outcomes (Barlett et al., 2024; Lee et al., 2026; C. Zhu et al., 2021). Based on these important contributions, it remains useful to consider that many existing syntheses combine traditional bullying and cyberbullying, merge victim and perpetrator roles, or include broad age ranges spanning childhood, adolescence, and emerging adulthood, which may make it more difficult to identify patterns specific to secondary-school adolescents. In addition, prior reviews have typically focused on either factors associated with adolescent cybervictimization or consequences, rather than examining both within a single, coherent theoretical framework. The present systematic scoping review therefore seeks to extend this body of work by (1) concentrating on secondary-school adolescents, with 12–17 years as the target age range, (2) organizing factors associated with cyberbullying victimization and the associated psychosocial and physical health outcomes within a socioecological perspective that distinguishes individual, family, school/peer, and online-context levels (Bronfenbrenner, 1979; Swearer & Espelage, 2004), and (3) synthesizing the most recent empirical studies to provide an up-to-date, developmentally focused overview of the evidence. This approach aims to offer a theory-driven synthesis that is directly relevant to secondary-school prevention and intervention efforts and may contribute to clarifying an area of the literature on adolescent cyberbullying victimization that remains only partially understood.

Taken together, these limitations highlight the need for a review that focuses specifically on secondary-school adolescents and integrates factors associated with cyberbullying victimization and the associated psychosocial and physical health outcomes within a coherent theoretical framework. The present systematic scoping review is novel in that it (a) concentrates on secondary-school adolescents, with 12–17 years as the target age range, (b) organizes associated factors and psychosocial and physical health outcomes across multiple socioecological levels (individual, family, school/peer, and online context), and (c) synthesizes the most recent empirical evidence using a systematic scoping approach. This design allows for a developmentally focused synthesis that is directly relevant to school-based prevention and intervention efforts.

Given the complexity of cyberbullying and its associations with adolescent well-being, a comprehensive analysis of associated factors and outcomes is essential. Although recent reviews have synthesized global evidence on cyberbullying and its associated variables, most have included mixed populations or focused exclusively on psychological outcomes, overlooking the distinctive social and media dynamics that characterize adolescents’ online interactions and peer relationships (Grama et al., 2024; Kasturiratna et al., 2025; Lee et al., 2026). Moreover, cyberbullying victimization manifests differently across developmental stages, underscoring the need for age-specific analyses. Cyberbullying victimization has been associated with risky internet and social media use. Research has reported associations involving frequent engagement with social networking sites, instant messaging platforms, and online gaming environments with voice or text chats, as well as excessive use, victimization, and loneliness (L. Chen et al., 2017; Doğan et al., 2025; Hinduja & Patchin, 2023; Marciano et al., 2020; Marín-Cortés et al., 2019). Insufficient social support has also been associated with cybervictimization (Ortega-Barón & Carrascosa, 2018). Other associated factors include deficits in socio-emotional skills, low empathy, poor resilience, and reduced life satisfaction (García et al., 2020; Marín-Cortés et al., 2019; Navarro et al., 2018; Zych et al., 2019). Family and environmental characteristics examined in relation to cybervictimization include inadequate social support, poor communication with supportive figures, and limited education regarding online risks (Marín-Cortés et al., 2019; Marinoni et al., 2026; Maurya et al., 2023; Worsley et al., 2019). Guidance on safe online navigation and balanced technology use has also been discussed in relation to adolescents’ exposure to harmful online behaviors (Marín-Cortés et al., 2019).

Importantly, cyberbullying victimization has been consistently associated with a wide range of adverse psychosocial outcomes in adolescents. The psychosocial difficulties examined in association with cyberbullying victimization extend beyond psychological distress to include substance use and suicidal behaviors (Extremera et al., 2018; Hinduja & Patchin, 2019; Marciano et al., 2020; C. Zhu et al., 2021). Meta-analytic evidence shows that cyberbullying victimization is moderately related to higher levels of depressive and anxiety symptoms in this age group (Hu et al., 2021; Molero et al., 2022). Longitudinal findings further indicate that cybervictimization predicts subsequent increases in mental health problems over time, suggesting that these difficulties are not merely concurrent correlates (Rose & Tynes, 2015). In addition, systematic review evidence points to an elevated risk of suicidal ideation and attempts among adolescent cybervictims compared with non-victimized peers (Buelga et al., 2022), underlining the potential severity of its psychological impact.

In this review, “factors associated with cyberbullying victimization” denotes individual and contextual characteristics examined in relation to variation in victimization, including sociodemographic characteristics, psychological symptoms and resources, family and peer circumstances, and patterns of digital engagement. The term does not assign an antecedent or causal role to these variables. Their interpretation as correlates, risk or protective factors, mediators, or moderators depends on the specific association examined, the study design, and the analyses reported. The distinction from psychosocial outcomes reflects the focus of the two review questions: associated factors characterize variation in victimization, whereas psychosocial outcomes describe adolescents’ functioning examined in relation to victimization. Consequently, a variable such as depressive symptoms may be relevant to both syntheses. Its inclusion in both categories does not establish whether it precedes or follows victimization, or whether the relationship is bidirectional.

For the purposes of this review, the term “psychosocial outcomes” encompasses four domains examined in relation to cyberbullying victimization: psychological and emotional functioning, including depressive and anxiety symptoms, loneliness, self-esteem, and life satisfaction; behavioral functioning, including externalizing problems, self-harm, and substance use; social and interpersonal functioning, including social self-efficacy and perceived social support; and academic functioning, including academic performance. Physical health outcomes refer to indicators of physical health and somatic well-being and are considered an additional outcome domain, distinct from the psychosocial domains for the purposes of this synthesis. This distinction concerns the constructs examined and does not imply that these domains are unrelated. The broader term “health and well-being” encompasses both psychosocial and physical health domains. Throughout the review, “outcomes” denotes variables examined in association with cyberbullying victimization and does not, by itself, imply temporal ordering or causality.

In this review, we draw on a socioecological perspective, which conceptualizes adolescent behavior as shaped by multiple, interconnected contexts at the individual, family, school/peer, and broader online-environment levels (Bronfenbrenner, 1979; Swearer & Espelage, 2004). Applied to cyberbullying, this framework guides the examination of associated factors across individual, family, school/peer, and online-context levels, without assuming that these factors precede victimization or that interactions between levels have been causally established. Accordingly, the present systematic scoping review focuses on identifying factors associated with adolescent cyberbullying victimization across these ecological levels and synthesizing the associated psychosocial and physical health outcomes.

Therefore, the present systematic scoping review aimed to provide a broader understanding of cyberbullying victimization and to identify variables that may be relevant to psychosocial and educational interventions addressing this issue. Specifically, this systematic scoping review aimed to (1) identify factors associated with adolescent cyberbullying victimization across individual, family, school/peer, and online-context levels and (2) synthesize the associated psychosocial and physical health outcomes. The review focused on secondary-school adolescents, with 12–17 years as the target age range. Given the exploratory and evidence-mapping nature of the review, no confirmatory hypotheses were formulated. Instead, the review was guided by the following research questions: (1) What factors are associated with adolescent cyberbullying victimization across individual, family, school/peer, and online-context levels? and (2) What psychosocial and physical health outcomes are associated with adolescent cyberbullying victimization?

## 2. Materials and Methods

This study was conducted as a systematic scoping review. In line with the principles of systematic review methodology, we developed an a priori protocol, specified predefined eligibility criteria implemented a structured search strategy across the selected information sources, and performed duplicate screening and systematic charting of the data. The scoping approach reflects the primary aim of the review, which was to map the breadth, characteristics, and gaps of the existing evidence rather than to generate a pooled quantitative estimate of effect. The review was planned and reported in accordance with the Preferred Reporting Items for Systematic Reviews and Meta-Analyses extension for Scoping Reviews (PRISMA-ScR), which is an official extension of the PRISMA statement specifically developed for systematic scoping reviews (Tricco et al., 2018).

The review has been conducted in accordance with the Joanna Briggs Institute (JBI) 2020 framework (Peters et al., 2020), an updated set of guidelines and tools designed for conducting systematic reviews and evidence synthesis, as well as the Preferred Reporting Items for Systematic Reviews and Meta-Analyses (PRISMA) (Page et al., 2021). Additionally, the PRISMA extension for Scoping Reviews (ScR guidance) has been incorporated to meet the specific reporting requirements for scoping reviews (Peters et al., 2020; Tricco et al., 2018). The PRISMA Scoping Checklist for Scoping Reviews (https://www.prisma-statement.org/scoping, accessed on 18 September 2026), is provided in the Supplementary Material Table S1. An a priori protocol was developed specifically for the present review and prospectively registered in PROSPERO (CRD42024618034) and the Open Science Framework (OSF) (https://osf.io/k27qv/?view_only=9f16afd780d1402f8m27377e6e4ccf42c, accessed on 25 November 2025). The eligibility criteria, search strategy, target population, and review objectives corresponded to those specified in the registered protocol. No deviations from the registered protocol occurred.

The PECOS framework is an essential tool for formulating a structured and clear research question, aimed at obtaining the necessary evidence for the study. The components of the PECOS question are as follows: the population under study (secondary-school adolescents experiencing cyberbullying victimization, with 12–17 years as the target age range), the exposure or factor of interest (factors associated with cyberbullying victimization), the comparison group (not necessary in this review, as the focus is on analyzing results within the same group), the outcomes to be investigated (cyberbullying victimization and the associated psychosocial and physical health outcomes and the study design (cross-sectional and longitudinal studies).

### 2.1. Search Strategy

The literature search was conducted through three platforms: EBSCOhost, ProQuest, and Web of Science (Clarivate Analytics, All Databases). All databases available within each platform were selected to maximize retrieval within these sources. The individual databases and indexes searched within each platform are listed in Table S2 of the Supplementary Materials. This multi-platform approach was consistent with the interdisciplinary scope of the review, encompassing individual, family, peer, school, and online-context factors associated with adolescent cyberbullying victimization and its psychosocial outcomes. The search terms included “Cyberbullying”, “Cybervictims”, “Risk factors”, “Consequences”, “Adolescents”, and “Victims”, which were derived from Medical Subject Headings (MeSH). The following search string was used [Cyberbullying OR cyber bullying OR cyber-bullying OR online harassment OR online bullying OR cybervictim* OR online victim* OR cybervictim OR cyber victim OR cyber-witness* AND teen* OR adolesc* OR minor* OR high schooler AND risk factors OR predispos* OR predic* OR vulnerability OR prevent* OR consequences OR psychological variables OR psychological factors OR character traits OR personal* traits OR mental health OR psychological health OR Social skills OR Soft skills OR interpersonal skills OR social competence OR emotional intelligence]. The search strategy was intentionally designed to be restrictive to focus the review exclusively on cyberbullying victimization.

Searches in EBSCOhost, ProQuest and Web of Science (Clarivate Analytics, All Databases) databases were conducted by two independent researchers (A.N.V. and R.M.P.H). All searches were reviewed by C.M.G-S., and R.M.L-G. Articles were initially screened by examining their titles and abstracts to exclude irrelevant studies. The remaining articles were then thoroughly reviewed for eligibility. Full texts of the relevant studies were subsequently obtained and evaluated according to the inclusion and exclusion criteria, leading to the final selection of articles for review. The PRISMA flowchart (Figure 1) illustrates the steps involved in the screening and selection process. The final search was completed on 1 August 2026.

### 2.2. Inclusion and Exclusion Criteria

The following inclusion criteria were established. These criteria included the selection of: (1) peer-reviewed empirical research articles using quantitative methods; (2) studies focusing on secondary-school adolescents, with 12–17 years as the target age range. This age range was selected to represent the developmental stage of middle-to-late adolescence, corresponding to the secondary education period, which is characterized by a significant increase in online social interactions. Studies including participants from primary school or university levels were excluded, as these populations differ in their developmental processes and social and educational contexts. When the exact age range was not clearly reported, studies were included if participants were identified as secondary-school students. In these cases, school level served as a contextual indicator of the target population, rather than confirmation that all participants were aged 12–17 years. Consequently, strict adherence to the target age range could not be verified for every included sample; (3) articles published from 2013 up to and including 1 August 2026; and (4) studies written in English. Articles from any country of publication were considered, provided that they primarily addressed the issues raised in this review. Exclusion criteria were as follows: (1) review articles or meta-analyses; (2) comments, editorials, case reports, letters, or meeting/conference abstracts; and (3) duplicate studies.

### 2.3. Search and Study Selection

Two reviewers independently screened all records at both stages (titles/abstracts and full texts). Disagreements were resolved through discussion and, when necessary, consultation with a third reviewer. Inter-rater reliability was assessed using Cohen’s κ. Agreement was substantial, with κ = 0.72 for title/abstract screening and κ = 0.75 for full-text screening. The search covered publications from 2013 up to and including 1 August 2026, a period that coincides with the global expansion of smartphones and social media platforms such as Instagram, WhatsApp, and Snapchat. During these years, mobile technologies surpassed traditional devices and became central to adolescents’ communication, peer interaction, and identity construction (Anderson & Jiang, 2018; Kowalski et al., 2014). This temporal delimitation allows the review to include studies that reflect the technological and social transformations that have reshaped adolescents’ online experiences, changes that earlier reviews could not fully capture as they were conducted in outdated digital contexts no longer representative of current adolescent practices.

The search was filtered to include only English-language articles and academic publications. A total of 2.287 papers were identified. Because of the extensive range of databases searched, a large number of duplicates were retrieved. After removing duplicates, 697 articles remained. Following a screening process based on topics, titles, and abstracts, 39 articles were selected. Many of these were subsequently excluded because they focused primarily on traditional bullying or did not examine cyberbullying victimization as their main objective. After applying the age eligibility criteria, articles with samples of school-aged adolescents outside the selected age range or grades were excluded, resulting in 24 articles. Figure 1 presents a flowchart of the article selection process.

### 2.4. Data Extraction and Quality Assessment

Data extraction was performed using a matrix developed by the research team, which was adapted from the framework proposed by JBI (Munn et al., 2018; Peters et al., 2015, 2020) to meet the established objectives. As shown below (see Table 1), this matrix includes key data from the studies. Table 2 and Table 3 summarize the information extracted from the studies.

The quality assessment was conducted by two independent researchers (A.N.V. and R.M.P.H.), who performed the initial analysis of the limitations and potential risks of bias in the selected studies. Inter-rater reliability was substantial, with Cohen’s κ = 0.79. To ensure the accuracy and objectivity of the process, any disagreements or uncertainties regarding the assessment were discussed with two additional reviewers (R.M.L-G. and C.M.G-S.), who independently evaluated the quality criteria and provided their perspectives on possible biases and limitations in the studies. This methodology was adopted to minimize the risk of individual errors and enhance the reliability of the process, ensuring that the quality assessment was as accurate and objective as possible.

Study quality and risk of bias were assessed using the Effective Public Health Practice Project (EPHPP) Quality Assessment Tool (Armijo-Olivo et al., 2012). This tool includes six dimensions: Selection Bias (systematic errors in participant selection when study groups are not representative of the general population or when participant selection is not random), Study Design (overall methodology, including whether the design is adequate to answer the research question), Confounders (whether studies have adequately identified, controlled for, and adjusted for extraneous variables), Blinding (the practice of keeping the researcher, participant, or both “blind” to certain aspects of the study to increase the objectivity of results), Data Collection Method (whether methods used to collect data include validated and reliable instruments to ensure the validity of the results), and Withdrawals and Dropouts (whether the study adequately managed participant losses and conducted an appropriate analysis of missing data).

Each dimension was rated as strong (score of 1), moderate (score of 2), or weak (score of 3). Additionally, the overall quality of each article was categorized as follows: Strong (no weak ratings), Moderate (one weak rating), or Weak (two or more weak ratings). See Table 4 for more details.

### 2.5. Data Synthesis

In line with the central aim of this systematic scoping review, the authors conducted a detailed analysis of the objectives, methodologies, and results of each included study. They also assessed the primary limitations, as well as the quality and risk of bias of each study (see Table 2, Table 3 and Table 4 for more details). Additionally, potential research gaps were identified to contribute to the advancement of the field and strengthen the existing scientific evidence base.

Initially, the lead author performed a study-by-study analysis to identify limitations and gaps in each study. In a subsequent phase, the lead author, together with two other authors (R.M.L-G. and C.M.G-S.), examined and discussed the relevance of the data obtained, based on a comparison with existing literature. This process aimed to analyze the primary risk characteristics of adolescent victims of cyberbullying in order to guide and improve future research in this area. Any discrepancies detected during the article review process were resolved through consultation with the senior author (R.M.L-G.).

The narrative synthesis is organized around the two review questions: factors associated with cyberbullying victimization (Section 3.4), and associated psychosocial and physical health outcomes and their contextual modifiers (Section 3.5). Within this organization, the individual level covers personal characteristics, symptoms, and behaviors; the family level covers family circumstances and relationships; the school/peer level covers peer experiences and academic functioning; and the online-context level covers internet and social media engagement. Placement reflects the construct and context examined, rather than the source of the report or the setting in which participants were recruited. Findings spanning levels are presented with their connections made explicit. Family variables that modify associations with individual outcomes are distinguished from outcomes experienced by adolescents. Perceived social support is described without assigning a specific family or peer source when this is not specified. This organization does not imply that the studies used multilevel statistical analyses.

The thematic organization of the synthesis does not establish temporal or causal ordering. Cross-sectional findings are interpreted as associations. In this review, risk and protective factors imply a higher or lower likelihood of a subsequent outcome, respectively; when temporal precedence is not supported, the variables are described as correlates. Mediators refer to variables examined within specified indirect pathways, whereas moderators refer to variables examined as modifying the strength or direction of an association. References to mediation and moderation concern the corresponding analyses reported in the primary studies and do not, by themselves, establish causal mechanisms. Longitudinal findings are interpreted in relation to measurement timing and the analyses conducted; longitudinal design alone does not establish causality. A variable may be presented under both review questions when it is relevant to both syntheses. Such overlap does not establish bidirectionality, which cannot be inferred from co-occurrence or an association reported in only one temporal direction.

The synthesis focuses on cyberbullying victimization. Perpetration is discussed only in relation to victimization and is treated as a distinct behavioral correlate or outcome. Findings concerning broader cyberbullying involvement are not treated as equivalent to victimization-specific findings.

Study design and methodological quality were considered qualitatively when interpreting the narrative synthesis, with reference to the EPHPP domain ratings and study-specific limitations presented in Table 4. These assessments contextualized concerns regarding participant selection, confounding, measurement, and follow-up. The quality ratings were not treated as numerical weights or as formal certainty ratings for individual synthesized associations. Overall conclusions were therefore framed in relation to the methodological limitations of the contributing studies.

## 3. Results

The results are organized into five sections: literature search and study characteristics; quality of selected studies and risk of bias; research gaps; individual and contextual factors associated with cyberbullying victimization; and psychosocial and physical health associations with cyberbullying victimization.

### 3.1. Literature Search and Study Characteristics

The studies included in this analysis are distributed across several countries: eight studies were conducted in China (Cao et al., 2025; Chang et al., 2019; Q. Chen et al., 2018; Jiang et al., 2024; Lin et al., 2023; Liu et al., 2023; Xu et al., 2024; Y. Zhu et al., 2021), four in Spain (Gámez-Guadix et al., 2013; Morea & Calvete, 2022; Rodríguez-Enríquez et al., 2019; Santos et al., 2021), two in the United States of America (USA) (Wright, 2016a, 2016b), two in Greece (Athanasiou et al., 2018; Tsitsika et al., 2015), one in Brazil (Wendt, 2021), one in Canada (Xiao & Shapka, 2025), one in Colombia (Cassiani-Miranda et al., 2022), one in Israel (Olenik-Shemesh & Heiman, 2017), one in Malta (Grech & Lauri, 2022), one in Romania (Grigore & Maftei, 2020), one in Sweden (Frisén & Berne, 2020), and one in Turkey (Güçlü-Aydogan et al., 2025).

Regarding study design, eight longitudinal studies (Cao et al., 2025; Gámez-Guadix et al., 2013; Lin et al., 2023; Morea & Calvete, 2022; Wright, 2016a, 2016b; Xiao & Shapka, 2025; Xu et al., 2024) and sixteen cross-sectional studies (Athanasiou et al., 2018; Cassiani-Miranda et al., 2022; Chang et al., 2019; Q. Chen et al., 2018; Frisén & Berne, 2020; Grech & Lauri, 2022; Grigore & Maftei, 2020; Güçlü-Aydogan et al., 2025; Jiang et al., 2024; Liu et al., 2023; Olenik-Shemesh & Heiman, 2017; Rodríguez-Enríquez et al., 2019; Santos et al., 2021; Tsitsika et al., 2015; Wendt, 2021; Y. Zhu et al., 2021) were included. Sample sizes ranged from 204 to 18.341 adolescents, with a higher proportion of male participants. Where age ranges were reported, participant ages ranged from 12 to 17.9 years. The total sample size across all studies was 64.700, comprising 29.108 female and 35.592 male adolescents.

In terms of the instruments used, there was considerable variability. Across the included studies, cyberbullying victimization was assessed using a range of cyberbullying-specific self-report instruments, such as the Cyberbullying Questionnaire—Victimization subscale (CBQ-V; Gámez-Guadix et al., 2013; Morea & Calvete, 2022; Santos et al., 2021), the Garaigordobil Cybervictimization Scale (GCS; Rodríguez-Enríquez et al., 2019), the European Cyberbullying Intervention Project Questionnaire (ECIPQ; Grigore & Maftei, 2020), the Revised Cyberbullying Inventory (RCBI; Jiang et al., 2024; Wendt, 2021), the Cyberbullying Scale (CBS; Güçlü-Aydogan et al., 2025; Xiao & Shapka, 2025), the Student Survey Questionnaire of Cyberbullying (SSQC; Olenik-Shemesh & Heiman, 2017), and the Cyberbullying Victimization Scale (CBV; Lin et al., 2023; Liu et al., 2023). In several studies, victimization was also measured using items adapted from broader bullying or electronic aggression measures, including the Electronic Bullying Questionnaire (EBQ; Xu et al., 2024) and the Forms of Bullying Scale (FBS; Cao et al., 2025). Mental health outcomes were most frequently assessed with validated depression measures such as the Beck Depression Inventory-II (BDI-II; Q. Chen et al., 2018; Grigore & Maftei, 2020; Y. Zhu et al., 2021), the Center for Epidemiologic Studies Depression Scale (CES-D; Liu et al., 2023; Morea & Calvete, 2022; Wright, 2016b), the Children’s Depression Inventory (CDI; Wendt, 2021), and the depression subscale of the Brief Symptom Inventory-18 (BSI-18; Gámez-Guadix et al., 2013; Santos et al., 2021). Anxiety and broader internalizing symptoms were captured using instruments such as the Multidimensional Anxiety Scale for Children (MASC; Wright, 2016b), the State–Trait Anxiety Inventory (STAI; Grigore & Maftei, 2020), the Screen for Child Anxiety Related Emotional Disorders (SCARED; Xu et al., 2024), the Youth Self-Report (YSR; Athanasiou et al., 2018; Tsitsika et al., 2015), the Social Anxiety Scale for Children Revised (SASC-R; Cao et al., 2025), and the Social Anxiety Scale for Adolescents (SAS-A; Jiang et al., 2024). Post-traumatic stress symptoms were typically assessed using the UCLA PTSD Index (Q. Chen et al., 2018; Y. Zhu et al., 2021) or the Davidson Trauma Scale (DTS; Cassiani-Miranda et al., 2022), and suicidality was measured through specific items (Q. Chen et al., 2018) or subscales on suicidal ideation and self-harm, such as the CES-D-SI (Cassiani-Miranda et al., 2022), the DSHI (Lin et al., 2023), and the NSSI scale (Liu et al., 2023).

In addition, many studies assessed psychosocial resources and adjustment, including self-esteem and body esteem (e.g., Body Esteem Scale for Adolescents and Adults, BESAA; Frisén & Berne, 2020; Olenik-Shemesh & Heiman, 2017; Self-Esteem Scale, SES; Lin et al., 2023), perceived social support (e.g., Child and Adolescent Social Support Scale, CASSS; Wright, 2016a; Multidimensional Scale of Perceived Social Support, MSPSS; Olenik-Shemesh & Heiman, 2017), resilience (e.g., Resilience Scale for Adolescents, READ; Santos et al., 2021; Child and Youth Psychological Resilience Scale, CYPRS; Güçlü-Aydogan et al., 2025), self-efficacy (e.g., Perceived Self-Efficacy Questionnaire, PSEQ; Olenik-Shemesh & Heiman, 2017; Self-Efficacy Questionnaire for Children, SEQ-C; Güçlü-Aydogan et al., 2025), self-disclosure (Revised Self-Disclosure Scale, R-SDS; Xiao & Shapka, 2025), peer attachment (e.g., Inventory of Peer Attachment, IPPA; Lin et al., 2023; Adolescent Attachment Questionnaire, AAQ; Y. Zhu et al., 2021), and family functioning (e.g., Family APGAR; Cassiani-Miranda et al., 2022). School-related adjustment was examined using measures of school engagement (e.g., Emotional Engagement subscale of the School Engagement Scale; Liu et al., 2023) and academic achievement or self-reported school performance (e.g., Athanasiou et al., 2018; Tsitsika et al., 2015). Broader health and behavioral outcomes included life satisfaction (e.g., Satisfaction With Life Scale, SWLS; Chang et al., 2019; Santos et al., 2021), substance use and risk behaviors (e.g., Adolescent Drug Abuse Inventory, ADAI; Gámez-Guadix et al., 2013; Youth Risk Behavior Survey; Cassiani-Miranda et al., 2022; problem drinking and gambling; Y. Zhu et al., 2021), and indicators of problematic or addictive technology use (e.g., Generalized Problematic Internet Use Scale-2, GPIUS-2; Gámez-Guadix et al., 2013; Smartphone Addiction Scale—Short Version, SAS-SV, and Internet Gaming Disorder Scale, IGDS; Cao et al., 2025). Overall, the measurement strategy across studies relied almost exclusively on self-report questionnaires, with substantial heterogeneity in the specific instruments used but a consistent emphasis on established, psychometrically supported scales to capture both cyberbullying victimization and its associated psychosocial correlates and consequences. Figure 1 presents the flow diagram of the study selection process, from identification and screening to the final inclusion of studies in the review. Table 2 presents the instruments employed in the selected studies.

Reported cyberbullying victimization percentages ranged from 3.9% to 45% among studies providing these data. These estimates were obtained using different operational definitions, measurement instruments, recall periods, and sampling procedures and should not be interpreted as standardized comparisons between countries or populations. Table 3 summarizes the objectives, methodology, and main findings of the included studies.

**Figure 1:**
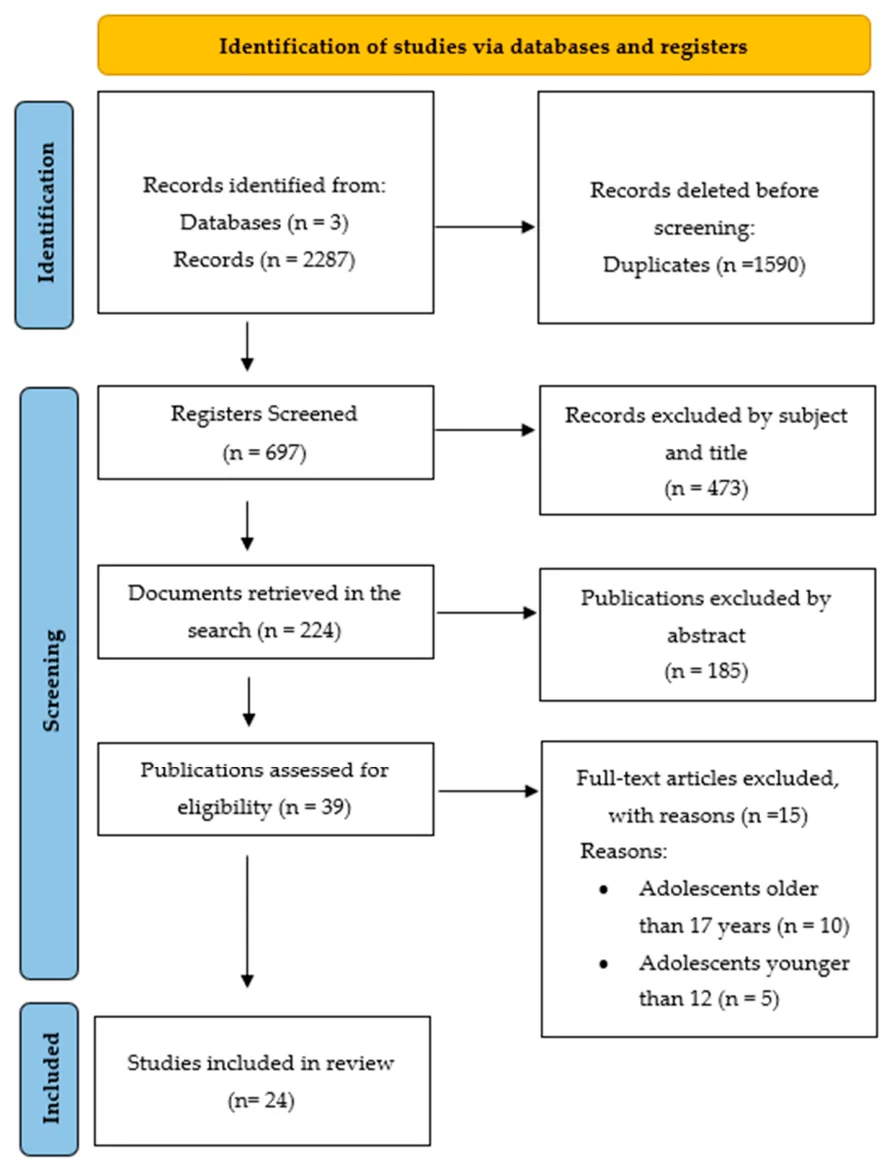
PRISMA flow diagram.

**Table 2:** Extraction of the instruments.

Study	Instruments
Gámez-Guadix et al. (2013)	The victimization subscale of the Cyberbullying Questionnaire (CBQ-V) The depression subscale of the Spanish version of the Brief Symptom Inventory (BSI 18) Adolescent Drug Abuse Inventory (ADAI) Generalized Problematic Internet Use Scale 2 (GPIUS-2)
Tsitsika et al. (2015)	Youth Self-Report (YSR) Self-report questionnaires on sociodemographic variables, Internet use, and cyberbullying
Wright (2016a)	Self-reported face-to-face victimization and cybervictimization (CV) The Child and Adolescent Social Support Scale (CASSS) Five-point Likert scale of assessing substance use
Wright (2016b)	Face-to-face victimization questionnaire Questionnaire assessing cyberbullying victimization Five-point Likert scale assessing parents’ involvement in their children’s digital media use The Center for Epidemiological Studies Depression Scale (CES-D) The Multidimensional Anxiety Scale for Children (MASC) The Revised University of California, Los Angeles Loneliness Scale (UCLA-LS)
Olenik-Shemesh and Heiman (2017)	The Student Survey Questionnaire of Cyberbullying (SSQC) The Body esteem scale for Adolescents and Adults (BESAA) The Multidimensional Scale of Perceived Social Support (MSPSS) The perceived self-efficacy questionnaire (PSEQ)
Athanasiou et al. (2018)	Self-completed questionnaires on sociodemographic data, internet usage characteristics, school achievement and parental control YSR
Q. Chen et al. (2018)	Chinese version of the 34-item Juvenile Victimization Questionnaire (JVQ) Items modified from the Abuse Assessment Screen (AAS) The 22-item self-report UCLA PTSD Index Chinese version of the Beck Depression Inventory II (BDI-II) The Chinese 12-item Short Form Health Survey (SF-12v2) Four-point Likert scale assessing adolescents’ suicidal ideation and deliberate self-harm
Chang et al. (2019)	Youth Sexuality Study of the Family Planning Association of Hong Kong Satisfaction With Life Scale (SWLS)
Rodríguez-Enríquez et al. (2019)	Questionnaire assessing sociodemographic characteristics Seven items adapted to assess smoking and drinking behaviors in adolescents Big Five Questionnaire for Children (BFQ-C) Garaigordobil Cybervictimization Scale (GCS)
Frisén and Berne (2020)	Self-Reported CB and CV BESAA Objectified body consciousness (OBC-Youth) Three subscales of the Sociocultural Attitudes Towards Appearance Questionnaire 4 (SATAQ-4)
Grigore and Maftei (2020)	Adapted version of BDI-II The State-Trait Anxiety Inventory (STAI) The short 9-item form of the European Cyberbullying Intervention Project Questionnaire (ECIPQ)
Santos et al. (2021)	Short version of the CBQ The Resilience Scale for Adolescents (READ) The depression subscale of the BSI The Spanish version of the SWLS
Wendt (2021)	Children’s Depression Inventory (CDI) Revised Cyberbullying Inventory (RCBI)
Y. Zhu et al. (2021)	The modified Relational Aggression Scale (RAS) Adolescent Attachment Questionnaire (AAQ) SF-12 Health Survey The Beck Depression Inventory–II (BDI-II) UCLA PTSD Adolescents’ self-reported problem drinking, cigarette smoking, and gambling involvement
Cassiani-Miranda et al. (2022)	Youth Risk Behavior Survey Questionnaire for high school students The Family APGAR questionnaire The WHO-5 questionnaire Brief Davidson Trauma Scale (DTS) The Center for Epidemiological Study on Depression’s Suicidal Ideation Scale (CES-D-SI)
Grech and Lauri (2022)	Section of the EUKids Online questionnaire
Morea and Calvete (2022)	The Changes, Cognitive Flexibility Test (CFT) A Spanish adaptation of the d2 Test CBQ CES-D
Lin et al. (2023)	18-item cyberbullying victimization scale (CBV) The self-esteem scale (SES) The Inventory of Peer Attachment Scale (IPPA) Deliberate Self- Harm Inventory (DSHI)
Liu et al. (2023)	Chinese version of the CBV CES-D The Emotional Engagement Subscale of the School Engagement Scale The Non-Suicidal Self-Injury scale (NSSI)
Jiang et al. (2024)	The revised Parental phubbing scale (RPPS) RCBI The revised Chinese version of the Social Anxiety Scale for Adolescents (SAS-A) Chinese self-control scale (C-SCS)
Xu et al. (2024)	The brief adaptation of the Electronic Bullying Questionnaire (EBQ) UCLA-LS-3 The Depression Self-Rating Scale for Children (DSRSC) The Screen for Child Anxiety Related Emotional Disorders (SCARED) Items from the Externalizing Behavioral Problems Scale (EEPE-AA) Self-reported items on cigarette smoking and alcohol abuse
Cao et al. (2025)	A six-item subscale derived from the Forms of Bullying Scale (FBS) The 14-item Cyberbullying Scale developed by Stewart and colleagues Fourteen items derived from the Social Anxiety Scale for Children Revised (SASC-R) The 10-item Smartphone Addiction Scale Short Version (SAS-SV) The nine-item scale suggested by the DSM-5 internet gaming disorder criteria (IGDS)
Güçlü-Aydogan et al. (2025)	Sociodemographic Questionnaire Turkish version of the Cyberbullying Scale (CBS), assessing exposure to cyberbullying victimization Child and Youth Psychological Resilience Scale (CYPRS) Self-Efficacy Questionnaire for Children (SEQ-C) Metacognitions Questionnaire for Children and Adolescents (MCQ-C)
Xiao and Shapka (2025)	The Revised Self-Disclosure Scale (R-SDS) Shapka and Law’s CBS Four items assessing time spent socializing online

**Table 3:** Characteristics of the included studies.

Author (Publication Year)	Country	Sample (Age Range)	Study Variables	Design	Main Findings: Associated Psychosocial and Physical Health Outcomes	Main Findings
Gámez-Guadix et al. (2013)	Spain.	N = 845 (*n* = 498 girls; *n* = 337 boys; *n* = 10 does not indicate sex). Age range = 13–17.	Cyberbullying and depressive symptoms, substance use and problematic Internet use.	Longitudinal.	Cybervictimization: 24.1% experienced one, 15.9% two, 8% three, and 4.7% four or more cyberbullying behaviors. Cybervictimization predicted depressive symptoms (r = 0.256, *p* < 0.001) and problematic Internet use (r = 0.197, *p* < 0.001) after 6 months.	No gender differences. More depressive symptoms and more substance use predicted more cyberbullying victimization after 6 months (0.11, *p* < 0.05 and 0.09, *p* < 0.05).
Tsitsika et al. (2015)	Greece.	N = 10,930 (*n* = 5719 girls; *n* = 5211 boys). Age range = 14–17.	Cybervictimization (CV), internalizing, externalizing and academic problems.	Cross-sectional.	Cybervictimization: 21.4%. Victims of cyberbullying reported lower academic performance (β = −0.06 [0.02], *p* < 0.001). Externalizing (β = 4.28 [0.24], *p* < 0.001) and internalizing (β = 4.54 [0.25], *p* < 0.001) problems were associated with CV.	Gender: Reports of CV were significantly (*p* < 0.001) more common among girls than boys (23.9% vs. 18.5%). CV was more prevalent among adolescents who used the internet and social media for two or more hours per day (OR = 1.52 [0.30–1.77], *p* <0.001).
Wright (2016a)	USA.	N = 867 (*n* = 442 girls; *n* = 425 boys). Age range = 13–15.	Perceived social support from parents, teachers and close friends as moderators of associations between Cybervictimization (CV) and substance use variables.	Longitudinal.	Cybervictimization: no data available. CV was negatively related to parental (β = −0.37, *p* < 0.001), teacher (β = −0.28, *p* < 0.01) and close friend social support (β = −0.31, *p* < 0.001) one year later; CV was positively associated with alcohol use (β = 0.51, *p* < 0.001) and illicit drug use other than marijuana (β = 0.32, *p* < 0.001) two years later; parental social support moderated the association between CV and adolescent substance use (β = 0.16, *p* < 0.01).	Gender: A multigroup comparison showed no gender differences; gender was therefore not included in the model.
Wright (2016b)	USA.	N = 568 (*n* = 301 girls; *n* = 267 boys). Age range = 13–15.	Parental mediation strategies in the association between cyberbullying victimization (CV) and psychosocial adjustment difficulties (depression, anxiety, loneliness) over one year.	Longitudinal.	Cybervictimization: no data available. CV was positively related to restrictive mediation, while it was negatively associated with shared parent–child mediation (β = −0.21, *p* < 0.05) and instructional mediation (β = −0.32, *p* < 0.001).	Gender: not disaggregated by sex.
Olenik-Shemesh and Heiman (2017)	Israel.	N = 204 (*n* = 98 girls; *n* = 106 boys). Age range = 14–16.	Cybervictimization (CV) in the context of body image, appearance, social support, and social self-efficacy. Body esteem, social support and social self-efficacy in cybervictims.	Cross-sectional.	Cybervictimization: 45%. High levels of CV were significantly correlated with low scores on body self-esteem (r = −0.29, *p* < 0.01), social support (r = −0.25, *p* < 0.01) and social self-efficacy (r = −0.159, *p* < 0.05).	No gender differences. Lower levels of body esteem and social support were associated with cybervictimization in the cross-sectional model [F(4, 203), Chi2(4) = 28.1, *p* < 0.001, R2 = 11.3%].
Athanasiou et al. (2018)	Greece, Germany, Iceland, Netherlands, Poland, Romania and Spain.	N = 13,708 (46.8% boys, 53.2% girls). Age range = 14 to 15.9 61.6%, 16 to 17.9 38.4%.	Cybervictimization and psychosocial, sociodemographic, and Internet use variables.	Cross-sectional.	Cybervictimization: 21.9%. The highest rates occurred in Romania (37.3%), Greece (26.8%) and Germany (24.3%), followed by Poland (21.5%).	Gender: Girls in the Netherlands were twice as likely to be cyberbullied as boys. Internalizing Problems: Greece (aOR = 1.04, *p* < 0.001), Spain (aOR = 1.06, *p* < 0.001), Romania (aOR = 1.05, *p* = 0.004), Poland (aOR = 1.03, *p* = 0.005), the Netherland (aOR = 1.04; *p* = 0.004) and Iceland (aOR = 1.05; *p* < 0.001). Externalizing problems: Greece (aOR = 1.04, *p* < 0.001), Spain (aOR = 1.04, *p* < 0.001), Romania (aOR = 1.02, *p* = 0.06), Poland (aOR = 1.05, *p* < 0.001), the Netherland (aOR = 1.03, *p* = 0.028) and Iceland (aOR = 1.07, *p* < 0.001). High parental family status: Iceland (aOR = 1.59; *p* = 0.048). Daily use of a social networking site (SNS) profile in Romania (aOR = 1.83 *p* = 0.004), Poland (aOR = 1.53; *p* = 0.028), and Germany (aOR = 1.66; *p* = 0.004). Daily internet use in Romania (aOR = 1.57 *p* = 0.041).
Q. Chen et al. (2018)	China.	N = 18,341 (53.3% boys; 46.7% girls). Age range: 15–17 years.	Cyberbullying and family victimization.	Secondary analysis of Chinese population survey (2009–2010).	Cybervictimization: 3.9%. Cybervictimization was positively correlated with PTSD (OR = 1.23; *p* < 0.001), Depression (OR = 1.05; *p* < 0.001), Physical health (OR = 0.95; *p* < 0.001), Mental health (OR = 0.99; *p* < 0.05), Deliberate self-harm and suicidal ideation (OR = 1.53; CI 95%: 1.228–1.914; *p* < 0.001).	Boys (30.6%) were more likely to report victimization than girls (28.8%) (*p* < 0.001). Family victimization (except child neglect) was associated with higher odds of bully victimization (OR = 1.99–5.36, *p* < 0.001). Parental divorce and separation (aOR = 1.27–1.68, *p* < 0.05), low family income (aOR = 1.11–1.35, *p* < 0.05), low educational attainment of the mother (aOR = 1.37–2, *p* < 0.05) and unemployment of the father (aOR = 1.43–2.13, *p* < 0.01) were associated with higher odds of cyberbullying victimization.
Chang et al. (2019)	China.	N = 3522 (57.4% boys; 42.6% girls). Grade = 7–12.	Cyberbullying victimization, life satisfaction (with family, classmates and academic results), suicidal ideation.	Cross-sectional study.	Cybervictimization: 11.9%. Suicidal ideation: Cyberbullying victimization was associated with suicidal ideation (OR = 2.48, 95% CI [1.99, 3.09]).	Life satisfaction was examined as a partial statistical mediator of the association between cyberbullying victimization and suicidal ideation (*b* = 0.17, SE = 0.04, 95% CI [0.10, 0.25]). An indirect association through satisfaction with family relationships was also reported (*b* = 0.19, 95% CI [0.12, 0.26]).
Rodríguez-Enríquez et al. (2019)	Spain.	N = 765 (*n* = 432 girls; *n* = 333 boys). Age range = 14–16.	Cybervictimization, personality, smoking and alcohol use; and gender-mediated differences.	Cross-sectional.	Cybervictimization: 39.9%.	Gender: Girls were more likely to be cyberbullied than boys (43.1% vs. 35.7%; *p* < 0.05). Higher extraversion (OR = 1.45; 95% CI = 1.19–1.78) and emotional instability/neuroticism (OR = 1.58; 95% CI = 1.32–1.89), and lower conscientiousness (OR = 0.73; 95% CI = 0.60–0.89) were significantly associated with higher risk of Cybervictimization. Alcohol consumption (OR = 1.99; 95% CI = 1.47–2.70) and tobacco consumption (OR = 2.55; 95% CI = 1.28–5.05) were associated with Cybervictimization.
Frisén and Berne (2020)	Sweden.	N = 480 (*n* = 233 girls; *n* = 247 boys). Age range = 13–15.	Appearance-related Cybervictimization and body-related concerns (body image, objectified body consciousness, and internalization of body ideals).	Cross-sectional.	Appearance-related Cybervictimization: 20.5%.	Body-related concerns: a more negative perception of general appearance (F = 6.91, *p* = 0.009, partial n^2^ = 0.02) and weight (F = 9.66, *p* = 0.002, partial n^2^ = 0.02); greater body shame (F = 7.16, *p* = 0.008, partial n^2^ = 0.02), greater internalization of the thin ideal (F = 7.76, *p* = 0.006, partial n^2^ = 0.02) and greater appearance-related media pressure (F = 10.03, *p* = 0.002, partial n^2^ = 0.02).
Grigore and Maftei (2020)	Romania.	N = 501 (*n* = 245 girls; *n* = 256 boys). Age range = 12–15.	State and trait anxiety, Cybervictimization, cyber-aggressiveness and depression.	Cross-sectional.	Cybervictimization: 18.6%. State anxiety was examined as a statistical mediator of the association between cybervictimization and depression in a cross-sectional model (0.39, SE = 0.07, 95% CI = [0.259–0.054]).	No gender differences. Positive associations were reported between trait anxiety (0.625; *p* < 0.001), state anxiety (0.785; *p* < 0.001) and depression (0.516; *p* < 0.001) and being a cybervictim.
Santos et al. (2021)	Spain.	N = 2108 (*n* = 1094 girls; *n* = 1014 boys). Age range = 12–17.	Resilience, Cybervictimization, depressive symptoms and life satisfaction.	Cross-sectional.	Cybervictimization: 10.83%. Cyberbullying victimization was associated with higher depressive symptom scores [(B) = 0.629, t(2101) = 14.878, *p* < 0.001, 95% CI = (0.546 to 0.712)] and reduced life satisfaction [B = −0.360, t(2101) = −9.966, *p* < 0.001, 95% CI = (−0.431 to−0.289)]. Resilience moderated the associations of cybervictimization with depressive symptoms and life satisfaction, with weaker associations at higher resilience levels [(B = −0.058, t(2101) = −11.690, *p* < 0.001, 95% CI = (−0.068 to −0.048]) and a smaller reduction in life satisfaction. [B = 0.052, t(2101) = 12.406, *p* < 0.001, 95% CI = (0.044 to 0.061)].	No gender differences.
Wendt (2021)	Brazil.	N = 268 (*n* = 136 girls; *n* = 132 boys). Age range = 13–17.	Specific depressive symptoms (anhedonia, ineffectiveness, interpersonal problems, negative mood and negative self-esteem) and cyberbullying victimization (CV).	Cross-sectional.	Cybervictimization: no data available. CV was positively associated with anhedonia [r = 0.29, *p* < 0.001], inefficacy [r = 0.28, *p* < 0.001], interpersonal problems [r = 0.26, *p* < 0.001], negative mood [r = 0.31, *p* < 0.001] and negative self-esteem [r = 0.14, *p* = 0.02].	Gender: not disaggregated by sex. Inefficacy (B = 0.46, *p* = 0.04) and negative mood (B = 0.37, *p* < 0.05) were significantly associated with cyberbullying victimization in the cross-sectional model.
Y. Zhu et al. (2021)	China.	N = 3232 (*n* = 1690 girls; *n* = 1542 boys). Age range = 15–17.	Cybervictimization (CV) and a range of health and mental health problems; parent–child attachment in these associations.	Cross-sectional secondary analysis.	Cybervictimization: 6.3% in the past year; 22.2% over their lifetime. CV was associated with higher odds (β = −1.58, *p* < 0.001; β = −2.22, *p* < 0.001), severe depressive symptoms (β = 3.74, *p* < 0.001; β = 4.48, *p* < 0.001) and symptoms of post-traumatic stress disorder/PTSD (β = 7.16, *p* < 0.001; β = 4.77, *p* < 0.001). CV was associated with an increase in the odds of lifetime problem drinking (OR = 1.64, *p* < 0.001), smoking (OR = 1.69, *p* < 0.001) and gambling (OR = 1.35, *p* < 0.05).	Gender: controlled for in the multivariate analyses.
Cassiani-Miranda et al. (2022)	Colombia.	N = 1462 (60.3% boys); 39.7% girls). Age range = 13–17.	Cybervictimization, demographic variables, general health status, family dysfunction, consumption (alcohol, cigarettes, cocaine and cannabis) and mental health risk (PTSD, depression and suicide).	Cross-sectional study.	Cybervictimization: 19.6%. Cybervictimization was associated with the risk of post-traumatic stress disorder (OR = 2.66; *p* < 0.001) elevated suicide risk (OR = 1.77; *p* < 0.001) and fair/poor health status (OR = 1.95; *p* < 0.001).	Female gender (OR = 1.68; 95% CI: 1.25–2.26; *p* = 0.001). Lifetime cigarette smoking (OR = 2.04; *p* < 0.001), lifetime alcohol drinking (OR = 1.78; *p* < 0.001), lifetime cannabis smoking (OR = 1.92; *p* < 0.001), lifetime cocaine smoking (OR = 3.05; *p* < 0.001) and family dysfunction (OR = 1.68; *p* = 0.005).
Grech and Lauri (2022)	Malta.	N = 367 (*n* = 191 girls; *n* = 169 boys; *n* = 7 other or did not answer). Age range = 13–16.	Cyberbullying, life satisfaction, well-being, self-harm, suicidal ideation.	Cross-sectional secondary analysis.	Cybervictimization: 24.5%. Cyberbullying victimization was associated with lower life satisfaction and well-being scores (t = −2.366, df = 353, *p* < 0.02). Self-harm: 18% of those who experienced cyberbullying Suicidal ideation: 30% of cybervictims.	Gender: not disaggregated by sex.
Morea and Calvete (2022)	Spain.	N = 698 (*n* = 285 girls; *n* = 413 boys). Age range = 12–17.	Cyberbullying victimization (CV) and executive functions.	Longitudinal.	Cybervictimization: no data available. CV was associated with higher depressive symptom scores (r = 0.31, *p* < 0.01) and greater cyberbullying perpetration 5–6 months later (r = 0.32, *p* < 0.01). The associations of CV with cyberbullying perpetration (B = 0.31, t(694) = 2.75, *p* = 0.006) and depression (B = 1.44, t(694) = 2.68, *p* = 0.007) were stronger when cognitive flexibility (CF) was low.	Gender: Selective attention among female adolescents is associated with higher levels of cyberbullying victimization.
Lin et al. (2023)	China.	N = 1368 (*n* = 547 girls; *n* = 821 boys). Grade = 10–11.	Self-esteem, cybervictimization (CV), non-suicidal self-harm (NSSI), and peer attachment.	Longitudinal.	Cybervictimization: no data available. CV was associated with lower self-esteem (β = −0.14, SE = 0.03, *p* < 0.001); lower self-esteem was associated with more frequent NSSI one year later (β = −0.05, SE = 0.02, *p* < 0.001). Peer attachment moderated the association between CV and self-esteem (β = −0.09, SE = 0.04, *p* < 0.05).	Gender: Used as a covariate.
Liu et al. (2023)	China.	N = 1006 (*n* = 521 girls; *n* = 485 boys). Age range = 12–15.	Cybervictimization (CV), non-suicidal self-harm (NSSI), depression and school connectedness (SC).	Cross-sectional.	Cybervictimization: no data available. CV was associated with depression (β = 0.27, SE = 0.03, t = 9.00, *p* < 0.001, 95% CI [0.21, 0.33]) and NSSI (β = 0.14, SE = 0.03, t = 4.59, *p* < 0.001, 95% CI [0.08, 0.20]); depression was examined as a statistical mediator of the association between CV and NSSI in the cross-sectional model (indirect effect = 0.09, SE = 0.02, 95% CI [0.06, 0.14]). SC moderated the association between CV and depression (β = 0.08, SE = 0.03, t = 2.64, *p* < 0.01, 95% CI [0.02, 0.14]), weakening the indirect association.	Gender: Used as covariate.
Jiang et al. (2024)	China.	N = 1034 (*n* = 543 girls; *n* = 491 boys). Age range = 12–15.	Parental phubbing, social anxiety (SA), self-control (SC), adolescent cyberbullying (CB), and cybervictimization (CV).	Cross-sectional.	Cybervictimization: 29.0% reported being involved in CB at least once, and 44.9% suffered from CV at least once.	Gender: controlled for in the multivariate analyses. Parental phubbing was positively associated with CB (β = 0.09, *p* = 0.004), self-control was negatively associated with CB (β = −0.32, *p* < 0.001), which is a significant positive predictor for CB (β = 0.15, *p* < 0.001). Parental phubbing was a significant positive statistical predictor of SA (β = 0.32, *p* < 0.001), which was a significant positive statistical predictor of CB (β = 0.15, *p* < 0.001). Parental phubbing and SA had predictive effects on CB at low self-control (β = 0.196, *p* < 0.001; β = 0.352, *p* < 0.001). The interaction terms between SC and parental phubbing (β = −0.08, *p* = 0.005) and between SC and SA were significant negative statistical predictors of CV (β = −0.20, *p* < 0.001) in adolescents.
Xu et al. (2024)	China.	N = 654 (*n* = 343 girls; *n* = 311 boys). Grades: 7–9.	Cyberbullying perpetration and victimization, loneliness, depressive symptoms, anxiety symptoms, delinquent behaviors, cigarette smoking and alcohol abuse.	Longitudinal.	Cybervictimization: no data available. Cyberbullying victimization showed a positive direct association with (ranging from b = 0.15 to b = 0.32, ps < 0.001) across Time 1 (T1; Grade 7) to Time 3 (T3; Grade 9), and significant effects on externalizing factors (b = 0.14, *p* < 0.01) only at T1. Additionally, cyberbullying victimization had a positive direct effect on anxiety symptoms (b = 0.10, *p* = 0.013) and alcohol abuse (b = 0.10, *p* = 0.006) at T1, as well as a direct effect on loneliness (b = 0.08, *p* = 0.014) at T3.	Gender: multi-group analyses showed non-significant sex differences.
Cao et al. (2025)	China.	N = 844 (*n* = 386 girls; *n* = 436 boys). Grade = 7–8.	Social anxiety symptoms (SAS), problematic smartphone use (PSU), Internet gaming disorder (IGD), offline peer victimization and cyberbullying victimization.	Longitudinal.	Cybervictimization: no data available.	Gender: boys, more than girls, who carried heavier burdens of childhood offline peer victimization displayed more SAS, which were associated with higher IGD, elevating their risk of being bullied online in adolescence (B = 0.161, SE = 0.103, 95% CI [0.007, 0.427], β = 0.018). The reported indirect pathway linked childhood peer victimization recalled at T1, social anxiety symptoms at T1 and T2, and cyberbullying victimization at T3 (B = 0.210, SE = 0.090, 95% CI [0.042, 0.397], β = 0.025).
Güçlü-Aydogan et al. (2025)	Turkey.	N = 574 (*n* = 357 girls; *n* = 217 boys). Grade = 9–12.	Psychological resilience, self-efficacy and metacognition.	Cross-sectional.	Cybervictimization: no data available.	Gender: not disaggregated by sex Negative meta-worry was positively associated with cyberbullying victimization scores in the cross-sectional regression analysis (β = 0.21, t = 3.86, *p* < 0.001; overall model: F(8, 573) = 17.06, *p* < 0.001).
Xiao and Shapka (2025)	Canada.	N = 354 (*n* = 161 girls; *n* = 193 boys. Age range = 13–16.	Sharing personal information online, socializing online, and cybervictimization.	Longitudinal.	Cybervictimization Scores: Cybervictimization among adolescents increased over time, with mean scores rising from 0.38 to 0.49. The study identified three different trajectories. A moderate-increasing trajectory (12.61% of the sample, intercept = 0.88, SE = 0.07, t = 11.38, *p* < 0.001; linear slope = 0.16, SE = 0.07, t = 2.13, *p* < 0.001); a low-increasing trajectory (83.90% of the sample, intercept = 0.18, SE = 0.02, t = 9.52, *p* < 0.001; linear slope = 0.08, SE = 0.01, t = 5.37, *p* = 0.033); and a high-decreasing trajectory (3.44% of the sample, intercept = 2.401, SE = 0.22, t = 10.68, *p* < 0.001; linear slope = −0.07, SE = 0.25, t = −2.60, *p* = 0.01).	Gender: Girls had 2.1 times lower odds of being in the moderate-increasing group compared to the high-increasing group; Girls had 2.32 times higher odds of being in the low-decreasing group compared to the moderate-increasing group. Students who shared personal information online (OR = 3.61) and spent more time socializing online (OR = 2.78) were more likely to belong to the moderate-increasing trajectory of cybervictimization compared with the other trajectories.

**Note:** Victimization, perpetration, and combined involvement estimates refer to distinct constructs. Unless mutually exclusive role categories were reported, victimization estimates may include adolescents who also perpetrated cyberbullying and should not be interpreted as estimates restricted to victimization-only profiles. **Abbreviations:** aOR = Adjusted odds ratio; CF = Cognitive flexibility; Chi2 = Chi-square; CI = confidence interval; CV = cybervictimization; df = Degrees of freedom; NSSI = Non-suicidal self-harm; OR = Odds ratio; PTSD = Post-traumatic stress disorder; SC = School connectedness; SE = Standard error; USA = United States of America.

### 3.2. Quality of Selected Studies and Risk of Bias

According to the criteria of the Effective Public Health Practice Project Quality Assessment Tool (EPHPP) (Armijo-Olivo et al., 2012), the quality of the studies analyzed was classified as high in four studies (Cao et al., 2025; Gámez-Guadix et al., 2013; Lin et al., 2023; Xu et al., 2024), moderate in thirteen studies (Chang et al., 2019; Q. Chen et al., 2018; Frisén & Berne, 2020; Jiang et al., 2024; Liu et al., 2023; Morea & Calvete, 2022; Rodríguez-Enríquez et al., 2019; Santos et al., 2021; Tsitsika et al., 2015; Wright, 2016a, 2016b; Xiao & Shapka, 2025; Y. Zhu et al., 2021), and low in seven studies (Athanasiou et al., 2018; Cassiani-Miranda et al., 2022; Grech & Lauri, 2022; Grigore & Maftei, 2020; Güçlü-Aydogan et al., 2025; Olenik-Shemesh & Heiman, 2017; Wendt, 2021). The reported limitations included the following: use of self-report measures, which may introduce biases (Athanasiou et al., 2018; Cao et al., 2025; Cassiani-Miranda et al., 2022; Chang et al., 2019; Q. Chen et al., 2018; Frisén & Berne, 2020; Grigore & Maftei, 2020; Güçlü-Aydogan et al., 2025; Jiang et al., 2024; Lin et al., 2023; Liu et al., 2023; Olenik-Shemesh & Heiman, 2017; Rodríguez-Enríquez et al., 2019; Santos et al., 2021; Tsitsika et al., 2015; Wendt, 2021; Wright, 2016b; Y. Zhu et al., 2021); cross-sectional design, preventing causal inferences from being drawn (Athanasiou et al., 2018; Cassiani-Miranda et al., 2022; Chang et al., 2019; Q. Chen et al., 2018; Frisén & Berne, 2020; Grech & Lauri, 2022; Grigore & Maftei, 2020; Güçlü-Aydogan et al., 2025; Jiang et al., 2024; Liu et al., 2023; Olenik-Shemesh & Heiman, 2017; Rodríguez-Enríquez et al., 2019; Santos et al., 2021; Tsitsika et al., 2015; Wendt, 2021; Y. Zhu et al., 2021); delays in data collection, affecting the timeliness of findings (Athanasiou et al., 2018; Q. Chen et al., 2018; Xiao & Shapka, 2025); time interval between waves, which may impact the interpretation of longitudinal trends (Xiao & Shapka, 2025); use of a limited number of items to measure key variables (Cassiani-Miranda et al., 2022; Chang et al., 2019; Q. Chen et al., 2018; Frisén & Berne, 2020; Xu et al., 2024); sample size constraints, reducing the generalizability of results (Olenik-Shemesh & Heiman, 2017; Xiao & Shapka, 2025); lack of control over traditional bullying, potentially confounding the analysis of cybervictimization (Frisén & Berne, 2020; Xu et al., 2024); absence of baseline data on cyberbullying victimization, limiting longitudinal assessments (Cao et al., 2025); measurement difficulties during the COVID-19 pandemic, which may have affected data accuracy (Güçlü-Aydogan et al., 2025); geographical and cultural limitations, restricting the applicability of findings across different populations (Lin et al., 2023; Liu et al., 2023). Not all world regions were equally represented among the studies included. Most were conducted in European and East Asian contexts, with fewer from the Americas and underrepresented regions such as Africa and Oceania. This uneven geographic distribution suggests that certain countries and cultural contexts are overrepresented, which may limit the cross-cultural generalizability of the review’s findings. Additional limitations included theoretical limitations, such as conceptual inconsistencies in defining cybervictimization (Rodríguez-Enríquez et al., 2019); exclusion of absent or out-of-school adolescents, which may overlook vulnerable populations (Tsitsika et al., 2015); failure to report study limitations, reducing transparency and replicability (Gámez-Guadix et al., 2013; Grech & Lauri, 2022). The methodological quality of the included studies and their risk of bias are summarized in Table 4.

**Table 4:** Quality of selected studies and risk of bias.

First Author (Publication Year)	Selection Bias	Study Design	Confounders	Blinding	Data Collection Method	Withdrawals and Dropouts	Global Assessment	Additional Biases
Gámez-Guadix et al. (2013)	Strong	Moderate	Strong	Moderate	Moderate	Moderate	Strong	Study limitations were not explicitly reported, which reduces transparency and hampers the reproducibility of the findings.
	Strong	Weak	Strong	Moderate	Moderate	Strong	Moderate	Cross-sectional design precludes causal inference; reliance on self-report may introduce response and social desirability bias; exclusion of adolescents who were absent or not enrolled on the survey day limits representativeness, and findings may not generalize to Europe as a whole or to other regions.
Tsitsika et al. (2015)	Strong	Moderate	Strong	Weak	Moderate	Strong	Moderate	Only three measurement points over a three-year period may not fully capture developmental changes in the key variables; the sample was restricted to high school students, who tend to report higher substance use than middle-school adolescents, which limits generalizability.
Wright (2016a)	Strong	Moderate	Strong	Weak	Strong	Strong	Moderate	Exclusive reliance on self-report may be affected by recall and social desirability bias; technology mediation and cyberbullying victimization were assessed only at baseline, preventing examination of temporal ordering and potential moderation effects.
Wright (2016b)	Moderate	Weak	Weak	Moderate	Strong	Moderate	Weak	Small sample size reduces statistical power; cross-sectional design precludes causal inference; exclusive use of self-report increases the risk of response and social desirability bias.
Olenik-Shemesh and Heiman (2017)	Strong	Weak	Weak	Moderate	Moderate	Strong	Weak	Cross-sectional design precludes causal inference; reliance on self-report may introduce reporting bias; data were collected only from students present on the survey day, which may affect representativeness; the time lag between data collection and publication may limit the relevance of the findings to current patterns of cybervictimization.
Athanasiou et al. (2018)	Strong	Weak	Strong	Moderate	Strong	Strong	Moderate	Self-report data may be affected by recall and social desirability bias; data were collected in 2010, so cyberbullying patterns may differ from current contexts; cyberbullying was assessed with a limited number of items, constraining measurement depth.
Q. Chen et al. (2018)	Moderate	Weak	Moderate	Moderate	Strong	Moderate	Moderate	Cross-sectional design precludes causal inference; the sample included only adolescents enrolled in full-time middle and high schools, which may restrict generalizability; suicidal ideation and life satisfaction were measured with single items, reducing reliability and construct coverage.
	Moderate	Weak	Strong	Moderate	Moderate	Moderate	Moderate	Cross-sectional design precludes causal inference; self-report measures may introduce response bias; cybervictimization was defined using a low-frequency threshold (‘sometimes’ in the past year), which may overestimate prevalence; relatively low levels of reported cyberbullying may bias results towards the null; the study did not distinguish pure victims from bully–victims.
Chang et al. (2019)	Moderate	Weak	Strong	Moderate	Strong	Moderate	Moderate	Cross-sectional design precludes causal inference; self-report measures may be influenced by response and social desirability bias; the sample was drawn from only four schools, limiting generalizability; several constructs examined in relation to cyberbullying were measured with single, non-validated items; offline bullying involvement was not assessed, which may confound associations.
	Strong	Weak	Weak	Strong	Strong	Strong	Weak	Reliance on self-report in the school setting may reduce willingness to disclose sensitive information and increase socially desirable responding; relevant correlates of adolescent cyberbullying identified in previous research were not assessed, limiting the completeness of the model.
Rodríguez-Enríquez et al. (2019)	Moderate	Weak	Strong	Moderate	Strong	Strong	Moderate	Cross-sectional design precludes causal inference; exclusive use of self-report may introduce response and social desirability bias; although large, the sample is not nationally representative, which limits generalizability.
	Moderate	Weak	Weak	Weak	Moderate	Moderate	Weak	Cross-sectional design precludes causal inference; self-report assessment may be influenced by recall and social desirability bias.
Frisén and Berne (2020)	Strong	Weak	Strong	Moderate	Strong	Strong	Moderate	Cross-sectional design precludes causal inference; reliance on retrospective self-report increases the risk of recall and social desirability bias.
Grigore and Maftei (2020)	Moderate	Weak	Weak	Moderate	Strong	Moderate	Weak	Cross-sectional design precludes causal inference; cyberbullying and physical and psychological complaints were assessed via self-report only, using single items and without corroboration from caregivers or health professionals, which limits diagnostic precision and may increase measurement error.
	Moderate	Weak	Weak	Moderate	Strong	Moderate	Weak	Study limitations were not explicitly reported, which reduces transparency and limits the interpretation and replicability of the findings.
Santos et al. (2021)	Moderate	Moderate	Weak	Moderate	Moderate	Moderate	Moderate	Only two executive functions and depressive symptoms were examined, restricting the range of cognitive and internalizing outcomes considered; executive functions were measured only at baseline, limiting insight into change over time; findings from a community sample may not generalize to clinical populations.
	Strong	Moderate	Strong	Moderate	Strong	Strong	Strong	The longitudinal observational design does not, by itself, establish causality; reliance on self-report may introduce response and social desirability bias; the sample consisted solely of Chinese adolescents, which may limit cross-cultural generalizability.
Wendt (2021)	Moderate	Weak	Strong	Moderate	Moderate	Moderate	Moderate	Cross-sectional design precludes causal inference; self-report data may be affected by social desirability bias; participants were junior high school students from a single Chinese province, which limits generalizability; potential moderating effects of school connectedness on the relationship between depression and non-suicidal self-injury were not examined.
Y. Zhu et al. (2021)	Strong	Weak	Moderate	Moderate	Strong	Moderate	Moderate	Cross-sectional design precludes causal inference; exclusive reliance on self-report from parents and adolescents may introduce shared-method bias; school and peer perspectives were not included, which may omit relevant contextual information.
	Strong	Moderate	Strong	Moderate	Strong	Strong	Strong	Traditional offline bullying was not controlled for, potentially confounding associations; the cyberbullying measure did not distinguish between specific forms of involvement; the second-order psychopathology model showed convergence problems, which may affect parameter accuracy; only a restricted set of psychopathological symptoms was examined.
	Strong	Moderate	Strong	Moderate	Strong	Moderate	Strong	The longitudinal observational design does not, by itself, establish causality; retrospective self-reports of childhood peer victimization may be affected by recall and social desirability bias; the sample was restricted to junior middle school students, limiting generalizability; some instruments require further construct validation in the Chinese context; the absence of baseline cybervictimization hampers examination of change over time; the six-month interval between waves may be insufficient to capture symptom trajectories; offline and cyberbullying perpetration were not assessed, despite their known overlap with victimization.
Cassiani-Miranda et al. (2022)	Moderate	Weak	Weak	Moderate	Strong	Moderate	Weak	Cross-sectional design precludes causal inference; web-based, self-report data collection may introduce selection, response and social desirability bias; data were collected during the COVID-19 pandemic, when fluctuating stress levels and restricted social contact may limit generalizability to other periods.
Grech and Lauri (2022)	Strong	Strong	Moderate	Moderate	Strong	Weak	Moderate	Small sample size limits statistical power and the stability of trajectory estimates; data were collected 5–8 years before publication, which may reduce relevance to current online environments; the study focused exclusively on cybervictimization and three risk factors (sharing personal information, online socializing and gender), omitting other important forms of online risk and potentially relevant predictors.

**Abbreviations:** CB = cyberbullying; COVID-19 = coronavirus disease in 2019; CV = cybervictimization; NSSI = non-suicidal self-harm.

### 3.3. Research Gaps

Research gaps concerning factors associated with adolescent cybervictimization highlight areas requiring further investigation to improve understanding of the phenomenon. This systematic scoping review identified several such gaps, particularly in methodological and contextual approaches.

Regarding methodological gaps, although several measurement scales have demonstrated validity and reliability, the use of inconsistent or diverse methods to assess cyberbullying complicates cross-study comparisons and hinders the consolidation of findings (see Table 2). Additionally, many studies fail to focus on specific developmental periods, instead grouping children, preadolescents, adolescents, and young adults together. This lack of specificity reduces the effectiveness of targeted interventions. The review’s focus on secondary-school adolescents, with 12–17 years as the target age range, was intended to support a developmentally focused synthesis relevant to this educational context. However, incomplete age reporting in some included studies limits the extent to which this focus can be regarded as a methodological strength.

Regarding contextual gaps, research has predominantly focused on urban settings, with relatively few studies examining rural contexts (Lin et al., 2023; Liu et al., 2023) or conducting direct comparisons between urban and rural environments. Furthermore, there is a notable absence of studies exploring differences among communities with varying socioeconomic levels or cultural traditions. This limitation may leave additional barriers faced by certain populations insufficiently understood and hinder the development of effective, culturally tailored intervention strategies.

Beyond documenting well-established associations, this review also contributes by systematically mapping research gaps that limit a deeper and more accurate understanding of factors associated with adolescent cybervictimization. Our synthesis shows that the field is still dominated by cross-sectional, self-report studies conducted in a relatively narrow set of cultural and educational contexts, with limited use of longitudinal, multi-informant or multilevel designs. Methodologically, few studies examine dynamic or cumulative risk, interactions between offline and online victimization, or the potential moderating role of protective factors and resilience processes. Contextually, important populations and settings remain underrepresented, including adolescents from low- and middle-income countries, rural areas, and minoritized or highly vulnerable groups. By making these gaps explicit, the present review not only consolidates current knowledge but also delineates priorities for future research, such as the need for longitudinal and cross-cultural studies, more nuanced measurement of digital contexts, and designs capable of disentangling complex pathways linking individual, family, school and online factors to cybervictimization.

### 3.4. Individual and Contextual Factors Associated with Cyberbullying Victimization

To address the first review question, the findings on factors associated with cyberbullying victimization are organized across individual, family, school/peer, and online-context levels. This organization describes the domains examined and does not establish that the associated factors preceded victimization.

#### 3.4.1. Individual Level

At the individual level, gender emerged as a sociodemographic variable associated with cybervictimization, although findings differed across studies. Among the eleven studies that examined gender differences in the frequency of cybervictimization, only four reported significant differences. Three studies reported that female participants were more frequently cyberbullied (Cassiani-Miranda et al., 2022; Rodríguez-Enríquez et al., 2019; Tsitsika et al., 2015), whereas one study found that male adolescents were more frequently targeted in cyberspace (Q. Chen et al., 2018).

Regarding personal characteristics, Rodríguez-Enríquez et al. (2019) found that high neuroticism and extraversion, along with low conscientiousness, correlated with the likelihood of being cybervictimized. Low psychological resilience was also associated with cyberbullying victimization (Güçlü-Aydogan et al., 2025).

Psychological symptoms were another group of individual correlates. Anxiety (Grigore & Maftei, 2020), social anxiety (Cao et al., 2025; Jiang et al., 2024), and depression (Gámez-Guadix et al., 2013; Grigore & Maftei, 2020) were associated with higher cybervictimization scores. In Wendt’s cross-sectional study (Wendt, 2021), depressive symptoms, including negative mood and feelings of ineffectiveness, were associated with cybervictimization. These findings do not establish whether the symptoms preceded or followed victimization (Wendt, 2021).

Body-related self-perceptions and perceived support were also examined. In relation to appearance-related cyberbullying, Olenik-Shemesh and Heiman (2017) reported associations of low body self-esteem and inadequate social support with cybervictimization. At the behavioral level, two studies indicated that substance use, particularly alcohol and tobacco consumption, was associated with a higher likelihood of cybervictimization among adolescents (Gámez-Guadix et al., 2013; Rodríguez-Enríquez et al., 2019).

#### 3.4.2. Family Level

At the family level, dysfunctional family circumstances were associated with cybervictimization in two studies (Cassiani-Miranda et al., 2022; Q. Chen et al., 2018). In particular, Q. Chen et al. (2018) reported an association between experiences of family victimization and adolescent cybervictimization (aOR = 2.24–5.36, *p* < 0.001). That study (Q. Chen et al., 2018), along with Tsitsika et al. (2015), also identified associations with low socioeconomic and educational status.

Parental digital behavior connected family circumstances with individual characteristics. Jiang et al. (2024) reported that parental phubbing (parental neglect due to excessive smartphone use) was associated with cyberbullying and cybervictimization, particularly among adolescents with social anxiety and low self-control. This finding is presented at the family level because the focal exposure concerns parental behavior, while social anxiety and self-control concern individual characteristics.

#### 3.4.3. School and Peer Level

Within the school/peer level, prior offline peer victimization was linked to cybervictimization through individual psychological symptoms. Cao et al. (2025) reported that adolescents who had experienced offline victimization were more prone to social anxiety symptoms, which were associated with subsequent cybervictimization. This longitudinal finding connects peer victimization with individual social anxiety and later victimization in the online context (Cao et al., 2025).

#### 3.4.4. Online Context

Internet and social media use were associated with cybervictimization in three studies (Athanasiou et al., 2018; Tsitsika et al., 2015; Xiao & Shapka, 2025). Athanasiou et al. (2018) linked daily internet and social media use with cybervictimization in countries such as Romania, Poland, and Germany. Similarly, Tsitsika et al. (2015) found that adolescents who spent more than two hours per day online without parental supervision were more likely to experience cyberbullying, linking online activity with family supervision.

Additionally, Xiao and Shapka (2025) reported that increased online socialization and frequent sharing of personal information over time were associated with cybervictimization. These findings are grouped within the online context because they concern adolescents’ patterns of digital engagement; the behaviors themselves were assessed at the individual level.

### 3.5. Psychosocial and Physical Health Associations with Cyberbullying Victimization

To address the second review question, the findings below summarize the psychosocial and physical health outcomes associated with cyberbullying victimization and the contextual factors examined in these associations. Individual outcomes are distinguished from family modifiers, school-related functioning, and behaviors and experiences situated in the online context.

#### 3.5.1. Individual Level

At the individual level, the reviewed studies reported physical, psychological, emotional, and behavioral associations with cybervictimization. In the physical health domain, several studies reported poorer overall physical health among victims (Cassiani-Miranda et al., 2022; Q. Chen et al., 2018; Y. Zhu et al., 2021).

Regarding psychological and emotional outcomes, cybervictimization was associated with internalizing problems (Athanasiou et al., 2018; Tsitsika et al., 2015; Xu et al., 2024). Multiple studies reported higher levels of loneliness (Xu et al., 2024), anxiety (Xu et al., 2024), depression (Q. Chen et al., 2018; Gámez-Guadix et al., 2013; Liu et al., 2023; Morea & Calvete, 2022; Santos et al., 2021; Wendt, 2021; Y. Zhu et al., 2021), and post-traumatic symptoms (Cassiani-Miranda et al., 2022; Q. Chen et al., 2018; Y. Zhu et al., 2021). In their longitudinal study, Xu et al. (2024) examined cyberbullying involvement in relation to general internalizing and externalizing dimensions and specific psychopathological symptoms.

Individual psychological variables were also examined in relation to these associations. Grigore and Maftei (2020) identified state anxiety as a statistical mediator of the association between cybervictimization and depression in a cross-sectional model, while Morea and Calvete (2022) found that cognitive flexibility moderated the longitudinal association between cybervictimization and depressive symptoms, with a stronger association among adolescents with lower cognitive flexibility.

Cybervictimization was also associated with self-harm (Q. Chen et al., 2018; Grech & Lauri, 2022), non-suicidal self-injury (NSSI) (Lin et al., 2023; Liu et al., 2023), and suicidal ideation (Chang et al., 2019; Q. Chen et al., 2018; Grech & Lauri, 2022). Furthermore, two studies reported an association with lower life satisfaction among adolescents (Grech & Lauri, 2022; Santos et al., 2021).

Body-related perceptions and social adjustment were examined in studies of harassment targeting appearance, weight, or other physical characteristics (Frisén & Berne, 2020; Olenik-Shemesh & Heiman, 2017). Frisén and Berne (2020) reported associations with psychological distress, a more negative body image, greater internalization of the thin ideal, and heightened body shame. Olenik-Shemesh and Heiman (2017) reported associations with lower self-esteem, reduced social self-efficacy, and decreased perceived social support.

Regarding behavioral outcomes, cybervictimization was associated with externalizing problems (Athanasiou et al., 2018; Tsitsika et al., 2015). Studies also reported associations with smoking (Cassiani-Miranda et al., 2022; Y. Zhu et al., 2021), alcohol consumption (Cassiani-Miranda et al., 2022; Wright, 2016a; Xu et al., 2024; Y. Zhu et al., 2021), illicit drug use (Wright, 2016a), and gambling (Y. Zhu et al., 2021). Behavioral findings concerning problematic internet use and subsequent cyberbullying perpetration are presented under the online context below.

#### 3.5.2. Family Level

At the family level, the findings concerned modifiers of associations with individual outcomes. Parent–child bonding was reported to moderate the association between cybervictimization and post-traumatic symptoms, with stronger bonding associated with lower symptom severity (Y. Zhu et al., 2021). Wright (2016a) similarly reported that adolescents who perceived higher levels of parental support showed a weaker association between cybervictimization and alcohol or drug use, excluding marijuana. These findings connect family relationships with psychological and behavioral outcomes experienced by adolescents; they do not describe changes in family functioning as an outcome of victimization.

#### 3.5.3. School and Peer Level

Within the school/peer domain, the reported academic finding concerned an association between cybervictimization and poorer academic performance (Tsitsika et al., 2015). This finding concerns adolescents’ functioning in the school context. The findings on social self-efficacy and perceived social support (Olenik-Shemesh & Heiman, 2017), presented above, concern social adjustment but are not attributed to a specific peer source here.

#### 3.5.4. Online Context

Within the online context, cybervictimization was associated with problematic internet use (Gámez-Guadix et al., 2013). Morea and Calvete (2022) also reported a longitudinal association between cybervictimization and subsequent cyberbullying perpetration. Here, perpetration represents a distinct behavioral outcome examined in relation to victimization. This association does not, by itself, identify a bully–victim subgroup or establish a generalized reciprocal process between the two roles.

Appearance-related experiences also connected individual perceptions with the online context. Alongside the body-image findings described above, Frisén and Berne (2020) reported greater perceived social pressure from social media among cybervictimized adolescents. These findings describe individual behaviors and experiences situated in digital environments.

## 4. Discussion

The purpose of this systematic scoping review was to explore and summarize the most recent evidence on cyberbullying victimization and to delineate variables relevant to psychosocial and educational interventions. Specifically, the review aimed to (1) identify factors associated with adolescent cyberbullying victimization across individual, family, school/peer, and online-context levels and (2) synthesize the associated psychosocial and physical health outcomes. The review focused on secondary-school adolescents, with 12–17 years as the target age range, and examined mediation and moderation where these were reported in the primary studies. Previous reviews have reported that broad age ranges make it difficult to determine whether victimization patterns differ across developmental stages (Kasturiratna et al., 2025; Lee et al., 2026). This educational focus was intended to support a developmentally focused synthesis, although incomplete age reporting prevents confirmation of age homogeneity. The inclusion of studies published from 2013 onward was intended to capture research reflecting the technological and social transformations shaping contemporary adolescents’ digital experiences.

The discussion is framed within an ecological–developmental perspective, which considers individual characteristics, family relationships, school climate, and broader social–digital environments (Bronfenbrenner, 1979; Bronfenbrenner & Morris, 2006; Swearer & Espelage, 2004). This framework organizes the interpretation of the evidence; it does not establish that interactions between ecological levels caused cybervictimization or its associated outcomes. Association refers to relationships between measured variables. Prediction is interpreted in relation to the study design and measurement timing; statistical prediction alone does not establish temporal precedence or causation. Mediation refers to the indirect associations examined in specified models, whereas moderation concerns variation in the strength or direction of an association. Neither establishes a causal mechanism by itself. Accordingly, Section 4.1 discusses individual and contextual factors associated with cybervictimization, Section 4.2 addresses associated psychosocial and physical health outcomes and the variables examined in these relationships, and Section 4.3 and Section 4.4 consider limitations and future research and practical implications.

### 4.1. Individual and Contextual Factors Associated with Cyberbullying Victimization

Regarding the first objective, this review identified individual and contextual factors associated with adolescent cyberbullying victimization. Their relevance to prevention requires consideration of the study designs supporting each association. Inclusion in this section does not imply that a variable preceded victimization or that modifying it would prevent victimization.

At the individual level, gender emerged as a sociodemographic variable examined in relation to victimization. However, the reviewed studies presented contradictory findings, reflecting a lack of consensus over the past decade (see Table 3). For example, Athanasiou et al. (2018) reported no significant gender associations in most participating European countries, whereas Q. Chen et al. (2018) found higher cybervictimization among boys than girls in China. Similarly, a previous meta-analysis by Lozano-Blasco et al. (2023) has shown inconsistency in findings regarding gender differences in cyberbullying victimization, emphasizing that gender-related vulnerability may depend on contextual norms, peer cultures, and digital practices rather than on gender alone.

From a psychological perspective, certain personality traits and low psychological resilience were associated with cybervictimization (Güçlü-Aydogan et al., 2025; Rodríguez-Enríquez et al., 2019). Among personality traits, neuroticism (emotional instability) was associated with cybervictimization (Rodríguez-Enríquez et al., 2019). Related work by Alonso and Romero (2017) and Bayat et al. (2021) has considered personality characteristics in bullying and cyberbullying involvement. Emotional distress and difficulties in emotion regulation have also been discussed alongside digital engagement (Erreygers et al., 2018; Torrente et al., 2014). However, the proposed sequence from personality traits to distress, coping through social media, and subsequent victimization should not be interpreted as established by these associations. Cybervictims also exhibited higher extraversion and lower conscientiousness in the cross-sectional study by Rodríguez-Enríquez et al. (2019). Earlier work by Peluchette et al. (2015) examined personality and risky social-network behaviors in relation to victimization. Their temporal roles require further examination.

Psychological and behavioral difficulties also emerged as correlates of cybervictimization. Internalizing problems were associated with victimization in the cross-sectional studies by Tsitsika et al. (2015) and Athanasiou et al. (2018), and depressive symptoms were associated with victimization in Wendt’s cross-sectional study (Wendt, 2021). These findings do not establish pre-existing vulnerabilities. Previous syntheses and studies have examined psychological symptoms in relation to cybervictimization (Guo, 2016; Kasturiratna et al., 2025; Marciano et al., 2020; Molero et al., 2022; Zhou et al., 2024), but the temporal evidence must be considered separately for each variable.

For depressive symptoms, Gámez-Guadix et al. (2013) reported reciprocal longitudinal associations: cybervictimization predicted subsequent depressive symptoms, and depressive symptoms predicted subsequent cybervictimization over six months. These findings support temporal reciprocity in that study, without establishing causality. Complementary longitudinal research cited in the Discussion, rather than included in the 24-study synthesis, also requires a differentiated interpretation. Calvete et al. (2026) reported reciprocal associations between cybervictimization and depressive symptoms, but NSSI did not predict subsequent changes in cybervictimization. NSSI should therefore not be grouped with depression as an established antecedent on the basis of that study.

For anxiety, Cao et al. (2025) examined a longitudinal model linking childhood offline peer victimization, social anxiety symptoms, and subsequent cybervictimization. This provides evidence concerning the direction examined in that model, rather than a general reciprocal relationship between anxiety and cybervictimization. Related literature has also examined social withdrawal, smartphone dependence, and peer relations (Lim, 2023). Such findings do not make these constructs interchangeable. Overall, a variable’s appearance under both review questions does not demonstrate bidirectionality.

From a behavioral perspective, externalizing problems were associated with cyberbullying victimization (Athanasiou et al., 2018; Tsitsika et al., 2015). The reviewed and related literature described associations involving excessive or problematic internet use (Athanasiou et al., 2018; Díaz-Vicario et al., 2019; Tsitsika et al., 2015; Xiao & Shapka, 2025), sharing personal information online (Peluchette et al., 2015; Rodríguez-Enríquez et al., 2019; Xiao & Shapka, 2025), and substance use (Gámez-Guadix et al., 2013; Pichel et al., 2022; Rodríguez-Enríquez et al., 2019). Behavioral difficulties and patterns of digital engagement have also been examined in relation to problematic internet and social media use (Alonso & Romero, 2021; Marcos et al., 2025). Psychoactive substance use and problematic internet use have been associated with cybervictimization (Zsila et al., 2018), while other research has examined problematic internet use alongside cyberbullying, coercive sexting, and grooming (Marcos et al., 2025). These findings describe related behaviors and experiences; they do not establish a sequence in which digital addictions, substance use, and victimization mutually reinforce one another. In the longitudinal study by Gámez-Guadix et al. (2013), substance use predicted subsequent cybervictimization, whereas cybervictimization predicted subsequent problematic internet use. The reverse associations were not significant. Thus, the temporal pattern differed across variables and should not be generalized into a common reciprocal cycle.

At the family level, several contextual variables were associated with cybervictimization, including family victimization, poor family communication and cohesion, and parental supervision of online activities. Family dysfunction was reported in association with cybervictimization (Cassiani-Miranda et al., 2022; Q. Chen et al., 2018). Earlier meta-analyses by Guo (2016), Grama et al. (2024), and Lozano-Blasco et al. (2024) have also examined family factors associated with adolescents’ victimization. Previous research by Machimbarrena et al. (2019) considered emotional distance, limited parental support, cohesion, and communication in family relationships. These findings do not establish that the family circumstances preceded victimization or explain how the associations arose. Parental phubbing was examined in relation to cybervictimization alongside adolescents’ social anxiety and self-control (Jiang et al., 2024), while related research has considered parental phubbing, parent–child relationships, and self-control in relation to problematic mobile phone use (Niu et al., 2020). These variables connect family and individual domains, but their co-occurrence does not demonstrate the proposed developmental sequence. Lower parental financial and/or educational resources were also associated with cybervictimization (Q. Chen et al., 2018; Tsitsika et al., 2015). Limited resources for supporting adolescents have been discussed as a possible explanation (Bazaga, 2022), which requires direct examination.

Parental supervision of internet use is another relevant family and online-context variable. Prior research has considered parental mediation, unrestricted internet use, and technological stress (Athanasiou et al., 2018; Díaz-López et al., 2020; Marinoni et al., 2026). The included studies reported associations between patterns of internet use and cybervictimization (Athanasiou et al., 2018; Tsitsika et al., 2015), while other literature has examined problematic digital engagement in relation to family, academic, and social functioning (Díaz-Vicario et al., 2019). These findings connect family circumstances with adolescents’ digital engagement and psychosocial functioning, without establishing their temporal sequence.

### 4.2. Psychosocial and Physical Health Associations with Cyberbullying Victimization

Regarding the second objective, this review synthesized psychosocial and physical health outcomes associated with adolescent cyberbullying victimization (Athanasiou et al., 2018; Cassiani-Miranda et al., 2022; Chang et al., 2019; Q. Chen et al., 2018; Frisén & Berne, 2020; Gámez-Guadix et al., 2013; Grech & Lauri, 2022; Grigore & Maftei, 2020; Güçlü-Aydogan et al., 2025; Lin et al., 2023; Liu et al., 2023; Morea & Calvete, 2022; Olenik-Shemesh & Heiman, 2017; Rodríguez-Enríquez et al., 2019; Santos et al., 2021; Tsitsika et al., 2015; Wendt, 2021; Wright, 2016a, 2016b; Xiao & Shapka, 2025; Xu et al., 2024; Y. Zhu et al., 2021). These variables were examined across psychological, behavioral, physical, social, family, and school-related contexts. Within an ecological–developmental framework (Bronfenbrenner, 1979; Swearer & Espelage, 2004), the synthesis distinguishes outcomes experienced by adolescents from contextual variables examined as mediators or moderators. The term “outcome” identifies the focus of the second review question and does not imply a demonstrated consequence when temporal ordering is unavailable.

At the individual level, in the physical health domain, the reviewed studies reported associations between cybervictimization and poorer overall physical health (Cassiani-Miranda et al., 2022; Q. Chen et al., 2018; Y. Zhu et al., 2021). These cross-sectional findings indicate that physical health problems were examined alongside psychosocial difficulties. They do not establish a decline in physical health following victimization or demonstrate that psychological symptoms mediated this association.

In the psychological domain, this systematic scoping review identified associations between cyberbullying victimization and emotional difficulties (Athanasiou et al., 2018; Tsitsika et al., 2015), consistent with the broader literature reviewed by Agustiningsih et al. (2024). Across the included studies, victimization was associated with anxiety, loneliness, depressive symptoms, and other mental health difficulties (Q. Chen et al., 2018; Gámez-Guadix et al., 2013; Liu et al., 2023; Morea & Calvete, 2022; Santos et al., 2021; Wendt, 2021; Xu et al., 2024; Y. Zhu et al., 2021). The evidence encompasses both concurrent and longitudinal associations, which require different interpretations.

With regard to anxiety, previous research has reported associations with cybervictimization (Agustiningsih et al., 2024; Rose & Tynes, 2015). Related literature has also considered social anxiety, school-related anxiety, and interpersonal skills (Delgado et al., 2019; Núñez et al., 2021; Torrente et al., 2014). In the included longitudinal study by Xu et al. (2024), cyberbullying victimization and perpetration were examined in relation to general internalizing and externalizing dimensions and specific symptoms. These findings should not be interpreted as demonstrating that social withdrawal alleviates anxiety or that loneliness is an inevitable later consequence. The different anxiety-related constructs and models also preclude a general claim of reciprocal associations with cybervictimization.

Similarly, our findings are consistent with previous syntheses and empirical studies linking cyberbullying victimization with depressive symptoms (Garaigordobil & Machimbarrena, 2019; Holfeld & Mishna, 2019; Molero et al., 2022; Rose & Tynes, 2015). As discussed in Section 4.1, reciprocal temporal associations between victimization and depressive symptoms were reported by Gámez-Guadix et al. (2013) and Morea and Calvete (2022), whereas cross-sectional associations alone cannot establish either direction. Grigore and Maftei (2020) examined state anxiety as a statistical mediator in the association between cybervictimization and depression. Because their study was cross-sectional, this indirect association does not establish that victimization first increased anxiety and that anxiety subsequently increased depression. Cybervictimization was also associated with post-traumatic symptoms in the cross-sectional study by Y. Zhu et al. (2021). Related longitudinal research by Holfeld and Mishna (2021) examined victimization and post-traumatic symptoms over time. These findings warrant attention to post-traumatic symptoms, without establishing that repeated victimization caused a progressive deterioration in mental health.

Regarding suicidal ideation and self-harm, the reviewed studies reported associations with cybervictimization (Chang et al., 2019; Q. Chen et al., 2018; Grech & Lauri, 2022), consistent with the wider literature on suicidal behavior (Buelga et al., 2022; Hinduja & Patchin, 2019; John et al., 2018; Winstone et al., 2024). Studies also examined self-harm and NSSI in relation to cybervictimization (Chang et al., 2019; Q. Chen et al., 2018; Faura-Garcia et al., 2021; Lin et al., 2023; Liu et al., 2023; Predescu et al., 2024; Winstone et al., 2024). These associations should not be interpreted as demonstrating that adolescents engaged in self-injury specifically to cope with victimization. Lin et al. (2023) examined a longitudinal model linking cybervictimization to NSSI through self-esteem, with peer attachment as a moderator. Liu et al. (2023) examined depression as a statistical mediator and school connectedness as a moderator in a cross-sectional model of the association between cybervictimization and NSSI. The role assigned to depression within that model does not establish its temporal position. Neither study provides a basis for treating NSSI as a demonstrated antecedent of cybervictimization. Earlier longitudinal evidence synthesized by Lee et al. (2026) supports examining mental health associations over time, but should not be generalized into an inevitable cycle of progressively worsening symptoms and repeated victimization.

Personal resources were examined as possible modifiers or explanatory variables in these associations. Santos et al. (2021) found that resilience moderated the associations of cybervictimization with depressive symptoms and life satisfaction in a cross-sectional study. Life satisfaction was an outcome in that study, rather than a mediator of the association with depression. In contrast, Chang et al. (2019) examined life satisfaction with family, classmates, and academic results within a cross-sectional mediation analysis of the association between cybervictimization and suicidal ideation. These statistical findings do not establish that increasing resilience or life satisfaction would reduce subsequent symptoms or suicidal ideation. Previous research has also considered these resources in relation to well-being and adjustment (Batmaz et al., 2023; Collen & Onan, 2021; García et al., 2020; Navarro et al., 2018), and the included studies examined their associations with victimization and mental health (Chang et al., 2019; Grech & Lauri, 2022; Güçlü-Aydogan et al., 2025; Santos et al., 2021).

Cognitive flexibility had a different analytical role. Morea and Calvete (2022) found that it moderated longitudinal associations between cybervictimization and subsequent depressive symptoms and perpetration, with stronger associations among adolescents with lower cognitive flexibility. This does not demonstrate that improving cognitive flexibility would prevent either outcome. Earlier literature has considered executive functions in behavioral regulation and problem-solving (Real Fernández et al., 2022), alongside personal resources relevant to cyberbullying (Güçlü-Aydogan et al., 2025). Their possible application in prevention and support requires direct evaluation.

In the behavioral domain, cybervictimization was associated with externalizing problems (Athanasiou et al., 2018; Tsitsika et al., 2015). Coping has been considered in the interpretation of these relationships (Holfeld & Mishna, 2019), but the associations do not establish the motives underlying particular behaviors. The included studies reported associations with substance use and gambling (Cassiani-Miranda et al., 2022; Wright, 2016a; Y. Zhu et al., 2021), while prior research examined subsequent substance use (Yoon et al., 2019) and co-occurring substance use among adolescents involved in cyberbullying (Pichel et al., 2022). Wright (2016a) reported longitudinal associations with alcohol and illicit drug use other than marijuana, and examined perceived social support as a moderator. The reported parental-support finding concerns variation in these associations; it does not demonstrate that support prevented adolescents from using substances to cope. The differing temporal findings across studies, including those reported by Gámez-Guadix et al. (2013), should not be combined into an assumed reciprocal relationship.

Furthermore, Morea and Calvete (2022) reported a longitudinal association between cyberbullying victimization and subsequent perpetration. Earlier meta-analytic work examined longitudinal associations between victimization and perpetration (Marciano et al., 2020), while Zhao et al. (2025) investigated a longitudinal mediation model involving personality functioning. These findings distinguish statistical prediction and modeled indirect associations from a demonstrated causal process of retaliation. They also indicate that victimization and perpetration roles may overlap (Morea & Calvete, 2022; Zhao et al., 2025). However, the temporal pattern was not uniform: Morea and Calvete (2022) reported that the association from perpetration to later victimization differed in direction across measurement intervals. This review did not provide a separate synthesis of adolescents who were both victims and perpetrators.

Another set of associations concerns self-perception and social adjustment. Across the included studies, adolescents experiencing appearance-related cybervictimization reported poorer body esteem, social self-efficacy, or perceived social support (Frisén & Berne, 2020; Olenik-Shemesh & Heiman, 2017). These cross-sectional findings do not demonstrate that victimization caused a distorted self-image. Previous research, including Halpern et al. (2017) and Ramos Salazar (2021), has also examined well-being, self-perception, and body-related variables in relation to cybervictimization. Related literature has considered self-esteem, social media, and exposure to appearance ideals (Nie et al., 2024; Rodríguez-Suárez et al., 2022; Singh & Dhillon, 2020), as well as eating-related behaviors and body dissatisfaction (de Diego Díaz Plaza et al., 2022; Ramos Salazar, 2021). These findings connect individual experiences with the social and digital context, but do not establish a causal sequence from cybervictimization through body dissatisfaction to eating disorders.

At the family level, parental mediation refers to parents’ approaches to adolescents’ digital media use; it should be distinguished from statistical mediation. Wright (2016b) examined restrictive, co-viewing, and instructive approaches in a longitudinal study. Cybervictimization was positively associated with restrictive mediation and negatively associated with co-viewing and instructive mediation. The study also examined whether these approaches moderated associations with later adjustment difficulties. These findings concern family characteristics and modifiers of individual outcomes, rather than changes in family functioning caused by victimization. Previous literature has considered family climate, communication, and parenting practices in relation to cyberbullying (García-Maldonado et al., 2011; Machimbarrena et al., 2019), but the reviewed associations do not demonstrate that a particular parenting approach prevents revictimization.

Finally, at the academic level, the cross-sectional study by Tsitsika et al. (2015) reported an association between cyberbullying victimization and poorer academic performance. Previous research by Gardella et al. (2017) and Martínez-Martínez et al. (2020) has also examined educational outcomes, while Ortega-Barón et al. (2016) considered school and family climate. These findings do not establish that victimization caused declining achievement or impaired later professional development. School connectedness, perceived support, self-esteem, and social skills have been examined in relation to psychological adjustment and self-harm (Holfeld & Baitz, 2020; Lei et al., 2020; Lin et al., 2023; Liu et al., 2023; Nie et al., 2024; Núñez et al., 2021; Saxsma et al., 2020). They identify areas for further assessment and intervention research, without demonstrating that modifying these variables will reduce victimization-related symptoms.

### 4.3. Limitations

The findings of this systematic scoping review should be interpreted in light of limitations affecting both the identification of relevant literature and the characteristics of the included studies. Independent screening and quality assessment helped reduce reviewer error and disagreement, but could not eliminate limitations arising from the coverage of the information sources and the eligibility restrictions applied.

First, searches were restricted to the databases and indexes available through EBSCOhost, ProQuest, and Web of Science. Although these sources were relevant to the interdisciplinary scope of the review, their selection does not guarantee comprehensive coverage of all relevant disciplinary and regional literature. Eligible studies indexed in other resources may therefore have been missed. Furthermore, the intentionally restrictive search strategy may have reduced retrieval of relevant studies using different terminology or indexing. These limitations may have influenced the range of associated factors, outcomes, and contexts represented in the synthesis. Consequently, areas with limited evidence in this review should be interpreted as gaps in the retrieved literature, rather than necessarily indicating an absence of research.

Second, eligibility was restricted to English-language, peer-reviewed quantitative studies published from 2013 up to and including 1 August 2026. The exclusion of non-English publications and grey literature introduces the possibility of language and publication bias, although the extent and direction of any resulting bias cannot be determined from this review. The exclusion of qualitative studies also limits the synthesis of adolescents’ perspectives and contextual experiences, while the publication window excludes earlier evidence. Together, these restrictions constrain the completeness and scope of the evidence map and should be considered when interpreting its findings and potential practical applications.

Sixteen of the 24 included studies were cross-sectional, limiting the identification of temporal relationships between cyberbullying victimization and its associated psychological and behavioral variables. These findings cannot establish whether such variables precede or follow victimization, and their classification as antecedents or consequences would therefore exceed the evidence available. The eight longitudinal studies provide information on associations over time, but their observational design does not, by itself, establish causality. Bidirectionality also requires direct examination of reciprocal temporal associations and cannot be inferred merely because a variable has been discussed as both a possible antecedent and an outcome. Statistical mediation and moderation findings must likewise be interpreted within the designs and assumptions of the primary studies. Cross-sectional indirect associations cannot establish temporal mediation, and a moderator does not by itself identify an effective intervention target.

Differences in the operational definitions and measurement of cybervictimization constrain comparability across studies. The behaviors covered by questionnaire items and the frequency thresholds used may influence which experiences are classified as victimization. For example, the use of a low-frequency threshold was identified as a limitation in Rodríguez-Enríquez et al. (2019), while some studies specifically examined appearance-related cybervictimization (Frisén & Berne, 2020; Olenik-Shemesh & Heiman, 2017). Recall periods also differed. Y. Zhu et al. (2021) reported victimization percentages of 6.3% for the preceding year and 22.2% over participants’ lifetimes within the same study. These figures refer to different observation periods and are not interchangeable prevalence estimates.

Heterogeneity also affected the assessment of associated outcomes. Studies used different instruments to measure specific symptoms, broader internalizing and externalizing dimensions, and other aspects of psychosocial and physical functioning (Table 2). Differences in construct coverage, scoring, and the use of brief items or multi-item measures limit direct comparisons. Furthermore, the correlations, regression coefficients, and odds ratios reported in Table 3 are not interchangeable estimates. Similar directions of association therefore do not establish equivalent magnitudes across studies.

Sampling differences further constrain interpretation. The studies varied in sample size, age and gender composition, and recruitment settings (Table 3). School-based recruitment could exclude adolescents who were absent or not enrolled, as noted in the limitations of the included studies (Athanasiou et al., 2018; Tsitsika et al., 2015), restricting representativeness. These sampling differences, together with variation in measurement and recall periods, mean that differences in reported victimization percentages cannot be attributed solely to countries or populations. The present synthesis does not isolate the relative contributions of methodological and contextual differences to this variability.

Methodological quality must also be considered when interpreting apparent agreement across studies. The EPHPP assessments in Table 4 identify study-level limitations concerning selection, confounding, measurement, and follow-up. An overall quality rating does not resolve differences in what was measured or establish the certainty of a particular association. Accordingly, the conclusions summarize the associations reported within this heterogeneous evidence base while distinguishing the temporal information available from different study designs. The review does not establish whether the same patterns would persist in a synthesis restricted to higher-quality studies.

Several included studies did not explicitly report participants’ age ranges (Cao et al., 2025; Chang et al., 2019; Güçlü-Aydogan et al., 2025; Lin et al., 2023; Xu et al., 2024). Although these studies identified participants by school grade or educational level, this information did not permit verification that all participants were within the target age range of 12–17 years. Thus, the review’s focus on secondary-school adolescents should not be interpreted as demonstrating age homogeneity across the included samples. This uncertainty limits the specificity of the conclusions for adolescents aged 12–17 years.

The review focused on factors and outcomes associated with cyberbullying victimization and did not provide a separate synthesis for adolescents who were both victims and perpetrators of cyberbullying. Consequently, it cannot establish how the vulnerabilities and outcomes of this subgroup differ from those of adolescents who experience victimization without engaging in perpetration. The findings should therefore not be interpreted as specific to victimization-only profiles. Future research should distinguish between victimization-only, perpetration-only, and combined victim–perpetrator profiles to clarify their respective associated factors and psychosocial and physical health outcomes.

Finally, the geographical distribution of the included studies was uneven. Eight of the 24 studies were conducted in China (Cao et al., 2025; Chang et al., 2019; Q. Chen et al., 2018; Jiang et al., 2024; Lin et al., 2023; Liu et al., 2023; Xu et al., 2024; Y. Zhu et al., 2021), accounting for one-third of the evidence base by study count. This concentration limits the cross-cultural generalizability of the synthesis, as findings from the settings represented cannot be assumed to apply uniformly to other countries or populations. The review therefore maps the evidence retrieved within its search scope and eligibility criteria rather than providing a geographically representative account of adolescent cyberbullying victimization. Further research in underrepresented settings is needed to assess the applicability of the identified associations across contexts.

### 4.4. Future Research and Practical Implications

The findings of this systematic scoping review provide a foundation for future research aimed at expanding knowledge of the factors associated with cybervictimization and exploring new prevention and support strategies for affected adolescents. In addition to summarizing reported associations, this review identifies and organizes research gaps that hinder a more precise understanding of factors associated with adolescent cybervictimization.

In addition, the results of the present review broadly converge with previous syntheses on cyberbullying victimization in adolescents, which have reported associations with internalizing difficulties, such as depressive and anxiety symptoms, as well as lower self-esteem and poorer psychosocial adjustment (Guo, 2016; Lei et al., 2020; Molero et al., 2022; Tran et al., 2023). At the same time, our results corroborate earlier work highlighting the relevance of school and peer contexts, including low perceived social support and negative classroom climate, as important correlates of cybervictimization (Gardella et al., 2017; Holfeld & Leadbeater, 2017; Ortega-Barón et al., 2016; Ragusa et al., 2024). In this sense, our review is consistent with the broader view that cyberbullying victimization is situated within a broader constellation of individual, relational, and contextual characteristics and experiences.

The contribution of this review lies primarily in integrating and organizing the available evidence on adolescent cyberbullying victimization across individual, family, peer/school, and online-context domains. It brings together associated psychosocial and physical health variables while identifying methodological limitations and gaps relevant to future research. This contribution should be understood as an evidence-mapping synthesis, rather than the identification of previously unknown determinants or the establishment of their relative importance.

Regarding future research directions, it is essential to further investigate gender-related patterns in cyberbullying and incorporate a gender-sensitive perspective in forthcoming studies. Although existing research has identified several associated factors among adolescents, further longitudinal studies are needed to examine their temporal relationships with victimization. Reciprocal associations should be tested directly for each variable rather than inferred from findings reported in separate directions. Longitudinal designs alone do not establish causality. The reviewed studies exhibit considerable methodological diversity, which constrains comparisons. Greater consistency in assessment tools and clearer reporting of intervention strategies would support comparability and the development of a stronger empirical foundation for future research.

With regard to sociodemographic variables, future research should also include studies that explicitly report or examine sexual orientation to broaden our understanding of its relationship with cyberbullying. Associations involving personality traits, low resilience, and limited family support require further investigation of their temporal ordering and the contexts in which they occur. Such research could inform the development and evaluation of prevention strategies tailored to family, school, and social environments, without assuming that the associated variables are causal intervention targets.

The available evidence does not support a reliable hierarchy of factors according to causal importance, predictive strength, or expected intervention benefit. Differences in study design, measurement instruments, and statistical analyses constrain comparisons between factors. Moreover, an association with cybervictimization does not establish that modifying the associated factor will reduce victimization or improve well-being.

For practical purposes, the domains identified in the review can nevertheless inform assessment and the selection of candidate outcomes for future intervention evaluations. Table 5 presents a non-ranked mapping of these domains, their possible applications, and candidate measurable outcomes based on variables examined in the reviewed literature. These applications are proposals for future evaluation, not interventions whose effectiveness has been established by this review.

Reported cyberbullying victimization could also be monitored across these applications. These measures are candidate evaluation outcomes, not established indicators of intervention success. The review does not determine an optimal intervention sequence, and the effectiveness of modifying these domains requires direct evaluation.

## 5. Conclusions

This systematic scoping review provides an integrated overview of factors associated with cyberbullying victimization and its psychosocial and physical health outcomes among secondary-school adolescents, with 12–17 years as the target age range. The findings indicate that cybervictimization is associated with individual characteristics, family dynamics, peer and school contexts, and patterns of online engagement. Across the reviewed studies, associations were reported with emotional difficulties, self-harm and suicidal behaviors, substance use, difficulties in self-perception and body image, poorer physical health, and impaired academic functioning.

The review also highlights associations involving personality traits, psychological symptoms, problematic internet use, family functioning, and parental mediation practices. An ecological–developmental perspective helps organize these individual and contextual associations. However, their identification across different levels does not establish that these factors preceded victimization or that interactions between them caused its onset or maintenance.

Sixteen of the 24 included studies were cross-sectional, preventing the establishment of temporal ordering in much of the evidence. The eight longitudinal studies provide information about associations over time, but their observational designs do not, by themselves, establish causality. Heterogeneity in measurement tools and limited reporting of key sociodemographic characteristics further constrain comparability and generalizability. Within these limitations, the synthesis consolidates and refines current knowledge and identifies domains requiring further investigation.

The findings can inform the development and evaluation of multilevel prevention and support strategies addressing individual, family, peer/school, and online-context factors. However, the review does not establish that modifying the associated factors will reduce cybervictimization or improve its associated outcomes. Continued research with longitudinal designs, standardized measures, and diverse samples, together with direct evaluations of interventions, is needed to clarify temporal relationships and inform psychosocial and educational initiatives aimed at protecting adolescents’ health and well-being.

## Figures and Tables

**Table 1:** Data extraction.

Extraction Fields	
Author(s)	Identification of the authors of the study to ensure proper acknowledgment of authorship and attribution.
Year of publication	Year of publication, to assess the timeliness and relevance of the information.
Source origin/country of origin	Contextualization of the results within a specific geographical setting.
Aims and purpose	The objective(s) of the research.
Study population and sample size	Description of the study population and sample size, when relevant, to evaluate the representativeness and scope of the findings.
Methodology and design	The methodology and design employed in the study, providing insight into the research approach.
How outcomes are measured	Methods used to measure the outcomes, which are important for evaluating the validity and reliability of the data.
Key findings related to the review question	Key findings of the study that contribute to answering the main research question.

**Table 5:** Potential applications and candidate measurable outcomes across domains associated with adolescent cyberbullying victimization.

Domain	Possible Application	Candidate Measurable Outcomes
**Individual**	Inform psychoeducational activities and individual or group support sessions addressing emotional difficulties and social-emotional resources.	Depressive and anxiety symptom scores; self-esteem and resilience scores.
**Family**	Inform family-focused work on communication, perceived support, and parental mediation of internet use.	Family communication and perceived support scores; reported parental mediation practices.
**Peer and school**	Inform school-based activities addressing peer integration and perceived support.	Perceived peer support scores; indicators of academic functioning.
**Online context**	Inform psychoeducation about internet use and sharing personal information online.	Problematic internet use scores; reported online information-sharing behaviors.

**Note.** The domains are presented without a hierarchy of importance. The applications and measurable outcomes are proposals for future evaluation based on variables covered in the review. They do not represent demonstrated intervention effects or establish an order of intervention priorities.

## Data Availability

The data that support the findings of this study are available on request from the corresponding author.

## Supplementary Materials

The following supporting information can be downloaded at: https://www.mdpi.com/article/10.3390/bs16101733/s1, Table S1: Preferred Reporting Items for Systematic Reviews and Meta-Analyses extension for Scoping Reviews (PRISMA-ScR) Checklist. Table S2: Search Documentation.
