# Supplementary material for: Factors and Psychosocial and Physical Health Outcomes Associated with Cyberbullying Victimization in Adolescents: A Systematic Scoping Review
Source: Behav Sci (Basel). 2026 Sep 23;16(10):1733. doi: 10.3390/bs16101733 (PMC13701436; doi:10.3390/bs16101733)
Supplement: Supplementary file 1 [file behavsci-16-01733-s001.zip › behavsci-4542239-supplementary.pdf]

Supplementary search documentation

Table S2. Search platforms, search-term selection, and Boolean structure

This table summarizes the search approach for the review of adolescent cyberbullying victimization and associated psychosocial and physical health outcomes. It distinguishes the terminology of the reported search expression from its implementation within individual databases.

A. Search platforms and coverage

Platform	Reported selection
**EBSCOhost**	All available databases.
**ProQuest**	All available databases.
**Web of Science**	All Databases.

Publication coverage: 2013 up to and including 1 August 2026. Final search date: 1 August 2026.

Reported search restrictions: English-language articles and academic publications. Eligibility was restricted to peer-reviewed empirical quantitative studies, including cross-sectional and longitudinal research, from any country.

Population eligibility: secondary-school adolescents, with 12–17 years as the target age range. Studies identifying participants as secondary-school students without reporting exact ages were also eligible; school level did not verify that every participant was aged 12–17 years.

Searches were conducted by A.N.V. and R.M.P.H. and reviewed by C.M.G-S. and R.M.L-G. Eligibility criteria are distinguished here from platform filters.

Selection and interpretation of terms

Search-term selection was informed by Medical Subject Headings (MeSH) terminology. The reported expression contains wording matching MeSH preferred headings and entry terms, together with additional keyword formulations, spelling variants, and truncated forms. Part B identifies these relationships, and Part C presents their conceptual organization.

A term's correspondence with a MeSH heading or entry term does not, by itself, establish that it was searched in an indexed subject-heading field. The source of the terminology and the database search fields are separate aspects of the strategy.

B. Reported terms and their relationship to MeSH

The classification below concerns the form of the terms in the reported expression. Matching capitalization is disregarded. Descriptor and entry-term matches are linked to the corresponding National Library of Medicine records.

Terminological category	Reported terms and relationship
**Wording matching preferred MeSH headings**	Cyberbullying; risk factors; mental health; Social skills; emotional intelligence.<br>Cyberbullying · Risk Factors · Mental Health · Social Skills · Emotional Intelligence
**Wording matching MeSH entry terms**	cyber bullying; online bullying → Cyberbullying. interpersonal skills; social competence → Social Skills.<br>Cyberbullying entry terms · Social Skills entry terms
**Truncated population keywords related to MeSH terminology**	teen*; adolesc*. These keyword forms relate to the Adolescent descriptor and its entry terms, including Teens, Teenagers, Adolescents, and Adolescence. The truncated strings are not themselves MeSH headings.<br>Adolescent descriptor and entry terms
**Additional cyberbullying keyword formulations**	cyber-bullying; online harassment; cybervictim*; online victim*; cybervictim; cyber victim; cyber-witness*.
**Additional population keyword formulations**	minor*; high schooler.
**Additional factor or outcome keyword formulations**	predispos*; predic*; vulnerability; prevent*; consequences; psychological variables; psychological factors; character traits; personal* traits; psychological health; Soft skills.

Note. Additional keyword formulations are reproduced as reported; they are not presented as an exhaustive assessment of their possible relationships to controlled vocabularies. All 31 reported formulations are retained. An entry term is an alternative access term associated with a descriptor, rather than a separate preferred heading.

MeSH terminology does not determine the review's eligibility age range. The target population was defined by the review criteria described in Part A.

C. Conceptual blocks and Boolean organization

Block	Terms as reported
**1 Cyberbullying and related terms**	Cyberbullying OR cyber bullying OR cyber-bullying OR online harassment OR online bullying OR cybervictim* OR online victim* OR cybervictim OR cyber victim OR cyber-witness*
**2 Adolescent population**	teen* OR adolesc* OR minor* OR high schooler
**3 Associated factors or outcomes**	risk factors OR predispos* OR predic* OR vulnerability OR prevent* OR consequences OR psychological variables OR psychological factors OR character traits OR personal* traits OR mental health OR psychological health OR Social skills OR Soft skills OR interpersonal skills OR social competence OR emotional intelligence

Conceptual combination: (Block 1) AND (Block 2) AND (Block 3).

In this conceptual presentation, MeSH-matching word forms and additional keyword formulations occur within the same OR lists; the three conceptual blocks are linked with AND. This does not document a separate combination of indexed controlled-vocabulary search lines and free-text search lines.

Expression reported in the manuscript

[Cyberbullying OR cyber bullying OR cyber-bullying OR online harassment OR online bullying OR cybervictim* OR online victim* OR cybervictim OR cyber victim OR cyber-witness* AND teen* OR adolesc* OR minor* OR high schooler AND risk factors OR predispos* OR predic* OR vulnerability OR prevent* OR consequences OR psychological variables OR psychological factors OR character traits OR personal* traits OR mental health OR psychological health OR Social skills OR Soft skills OR interpersonal skills OR social competence OR emotional intelligence]

Note. The expression above is reproduced with spacing normalized. The block structure clarifies its conceptual content; the expression is not a database-specific search-history export.
